# Composition and yield of non-cellulosic and cellulosic sugars in soluble and particulate fractions during consolidated bioprocessing of poplar biomass by *Clostridium thermocellum*

**DOI:** 10.1186/s13068-022-02119-9

**Published:** 2022-02-28

**Authors:** Ajaya K. Biswal, Neal N. Hengge, Ian M. Black, Melani A. Atmodjo, Sushree S. Mohanty, David Ryno, Michael E. Himmel, Parastoo Azadi, Yannick J. Bomble, Debra Mohnen

**Affiliations:** 1 Department of Biochemistry and Molecular Biology, University of Georgia, Athens, GA 30602 USA; 2 Complex Carbohydrate Research Center, University of Georgia, 315 Riverbend Rd, Athens, GA 30602 USA; 3 Biosciences Center, National Renewable Energy Laboratory, Golden, CO 80401 USA

**Keywords:** Consolidated bioprocessing, *Populus*, *Clostridium thermocellum*, Cellulose, Lignin, Pectin, Hemicellulose (xylan), Non-cellulosic wall polysaccharides

## Abstract

**Background:**

Terrestrial plant biomass is the primary renewable carbon feedstock for enabling transition to a sustainable bioeconomy. Consolidated bioprocessing (CBP) by the cellulolytic thermophile *Clostridium thermocellum* offers a single step microbial platform for production of biofuels and biochemicals via simultaneous solubilization of carbohydrates from lignocellulosic biomass and conversion to products. Here, solubilization of cell wall cellulosic, hemicellulosic, and pectic polysaccharides in the liquor and solid residues generated during CBP of poplar biomass by *C. thermocellum* was analyzed.

**Results:**

The total amount of biomass solubilized in the *C. thermocellum* DSM1313 fermentation platform was 5.8, 10.3, and 13.7% of milled non-pretreated poplar after 24, 48, and 120 h, respectively. These results demonstrate solubilization of 24% cellulose and 17% non-cellulosic sugars after 120 h, consistent with prior reports. The net solubilization of non-cellulosic sugars by *C. thermocellum* (after correcting for the uninoculated control fermentations) was 13 to 36% of arabinose (Ara), xylose (Xyl), galactose (Gal), mannose (Man), and glucose (Glc); and 15% and 3% of fucose and glucuronic acid, respectively. No rhamnose was solubilized and 71% of the galacturonic acid (GalA) was solubilized. These results indicate that *C. thermocellum* may be selective for the types and/or rate of solubilization of the non-cellulosic wall polymers. Xyl, Man, and Glc were found to accumulate in the fermentation liquor at levels greater than in uninoculated control fermentations, whereas Ara and Gal did not accumulate, suggesting that *C. thermocellum* solubilizes both hemicelluloses and pectins but utilizes them differently. After five days of fermentation, the relative amount of Rha in the solid residues increased 21% indicating that the Rha-containing polymer rhamnogalacturonan I (RG-I) was not effectively solubilized by *C. thermocellum* CBP, a result confirmed by immunoassays. Comparison of the sugars in the liquor versus solid residue showed that *C. thermocellum* solubilized hemicellulosic xylan and mannan, but did not fully utilize them, solubilized and appeared to utilize pectic homogalacturonan, and did not solubilize RG-I.

**Conclusions:**

The significant relative increase in RG-I in poplar solid residues following CBP indicates that *C. thermocellum* did not solubilize RG-I. These results support the hypothesis that this pectic glycan may be one barrier for efficient solubilization of poplar by *C. thermocellum*.

**Supplementary Information:**

The online version contains supplementary material available at 10.1186/s13068-022-02119-9.

## Background

We are at an inflection point in which a resource depletion, fossil fuel-based economy must be replaced by a sustainable carbon-negative bioeconomy [1, 2]. This transition includes the use of biomass as the major carbohydrate-rich renewable resource for production of transportation fuels, chemicals, and materials. Dedicated cellulosic feedstocks, such as trees and herbaceous grasses, along with residues from agricultural, forest restoration, and municipal solid waste are available at the scales needed for this transition. The development of large scale and cost-effective processing technologies to convert the structurally complex plant biomass to bioproducts, however, remains challenging [3].

The bulk of cell wall biomass in dedicated cellulosic feedstock is comprised of cellulose microfibrils, hemicelluloses, and lignin; with lesser amounts of pectin and glycoproteins [4–7]. The primary, secondary, tertiary, and quaternary structures of these components [8] and their final associations and architecture in the cell wall have evolved to vary by cell type and developmental state, thus providing sessile plants and their cells structural and functional properties that enable survival in harsh biotic and abiotic environments. The primary challenge for the use of plant biomass as a resource for the production of biofuels, chemicals and biocommodities is therefore the development of processing strategies that overcome natural recalcitrance to deconstruction and provide high yields of the sugars for fermentation [1, 3].

While transportation fuel for light duty vehicles is expected to be replaced by electric energy, liquid fuel will be required into the foreseeable future for aviation and marine transportation. Thus, efforts to produce liquid fuel from biomass remain critical. Multiple biomass processing and fermentation strategies to obtain C5 and C6 sugars from lignocellulosic biomass have been developed, including thermal, chemical, biochemical and microbial processes [3]. However, none have proven sufficiently cost-effective to yield a thriving biofuel industry. The biochemical approach has the potential advantage of lower capital costs and high conversion yields, however hurdles include the cost of pretreatment to “loosen” biomass structure and allow hydrolytic enzymes to gain access to cell wall polymers, the cost of exogenous enzymes needed in classical simultaneous saccharification and fermentation (SSF), and the production of inhibitors of the fermentation process during biomass pretreatment. An approach that circumvents some of these obstacles is consolidated bioprocessing (CBP), in which a suitable microorganism can both produce enzymes able to solubilize sugars from the untreated biomass and ferment the sugars to biofuels, providing a single step process. An optimized CBP platform would eliminate the need for exogenous enzymes and pretreatment, improve efficiency, and decrease capital investment, advances important for development of a cost competitive process [1, 3].

A leading microbial candidate for CBP is the anaerobic Gram-positive thermophile, *Clostridium thermocellum*, which is one of the most effective crystalline cellulose utilizing microbes found in nature [3]. CBP using *C. thermocellum* offers distinct process advantages, including reduced risk of microbial contamination and increased solubilization of biomass polymers due to high growth temperatures, facile integration into existing industrial processing schemes due to reduced cooling requirements, and the production of highly efficient cellulosomes which provide unique solubilization properties on crystalline cellulose [3]. *C. thermocellum* DSM1313 can hydrolyze all three major polysaccharide classes of cell wall polymers, cellulose, hemicellulose (e.g., xylan) and pectin; the latter being a major component of the “glue” that holds the cells of plant biomass together [9–11]. Limitations in the use of *C. thermocellum* for the production of biofuels include the inability of native strains to utilize C5 sugars and a low tolerance to ethanol [12], although progress has been made in identifying mutants tolerant to relatively high concentrations of ethanol [13].

Although *C. thermocellum* is very efficient at hydrolyzing and utilizing cellulose, and produces enzymes that solubilize the other two classes of non-cellulosic cell wall polysaccharides, hemicellulose and pectin, it has been reported that *C. thermocellum* cannot utilize the non-cellulosic polymers for growth [12]. Numerous studies have confirmed that wild-type *C. thermocellum* can hydrolyze xylan, the most abundant non-cellulosic polysaccharide in grasses and hardwood feedstocks, but cannot utilize xylose or arabinose for growth. There are fewer published studies on the metabolism of pectin by *C. thermocellum* and those that are available are contradictory. Aburaya and coworkers [14] reported that *C. thermocellum* cannot grow on pectin, whereas Spinnler and coworkers [15] showed that although *C. thermocellum* could not grow on monogalacturonic acid, a major glycosyl residue of pectin, it did grow well on polygalacturonic acid and sugar beet pectin. These contradictory results leave open the question as to whether or not *C. thermocellum* utilizes pectin.

*C. thermocellum* is highly effective in solubilizing pure crystalline cellulose, with reports of up to 100% solubilization of Avicel™ [3]. The solubilization of the cellulose and non-cellulosic cell wall polymers in plant biomass by *C. thermocellum* is more difficult due to the heterogeneous nature of these polymers and their homo- and hetero-associations. The degrees of solubilization of biomass by *C. thermocellum* depends on the type of biomass, severity of the pretreatment, and methods used to quantify carbohydrate yields. For example, total carbohydrate fractional yields of 20–31% and 32–45% have been reported for *C. thermocellum* solubilization of milled non-pretreated poplar and switchgrass biomass, respectively [16]. Pretreatment of biomass can increase solubilization rates and amounts dramatically; however, these processes require additional energy and processing steps and therefore increase process cost. The methods generally used to calculate mass fraction solubilization are based on fractional glucose, xylose, and arabinose yields following microbial growth and fermentation. In such calculations, changes in the other non-cellulosic sugars are typically not monitored since they represent only a small fraction of the sugars in the bioenergy dedicated grass and woody feedstock. For example, galacturonic acid, the major pectic sugar, accounts for only ~ 6% of poplar wood and 1% of switchgrass tillers [17]. However, although a minor component, changes in the amount and structure of pectin have been shown to increase both grass and woody biomass yield and to make the biomass more amenable to enzymatic deconstruction, due to the role of pectin in polymer/polymer and thus cell/cell adhesion in the wall [4]. Thus, knowledge of the efficiency of *C. thermocellum* in solubilizing and utilizing pectin during CBP is important for understanding and potentially improving the CBP process.

The goal of this study was to better understand the solubilization of cellulose and non-cellulosic sugars from milled, non-pretreated Center for Bioenergy Innovation (CBI) reference poplar [18] during CBP by *C. thermocellum* DSM1313. Determination of cellulosic and non-cellulosic sugar mass yields in both the fermentation broth and solid residues during CBP by *C. thermocellum* was carried out to identify potential cell wall structural and architectural changes and limitations to solubilization of non-pretreated poplar biomass. The results of this study provide information about non-cellulosic sugars and likely corresponding polymers that are not effectively solubilized from milled, non-pretreated poplar by *C. thermocellum.* This study also provides a foundation for comparisons of the efficacy of *C. thermocellum* solubilization of biomass from feedstock mutants and variants with modified cell wall and yield properties; as well as the effects of different biomass pretreatment strategies on the ability of *C. thermocellum* to solubilize biomass at different biomass loadings.

## Results

### Solubilization of poplar biomass using *C. thermocellum*

**Fig. 1:**
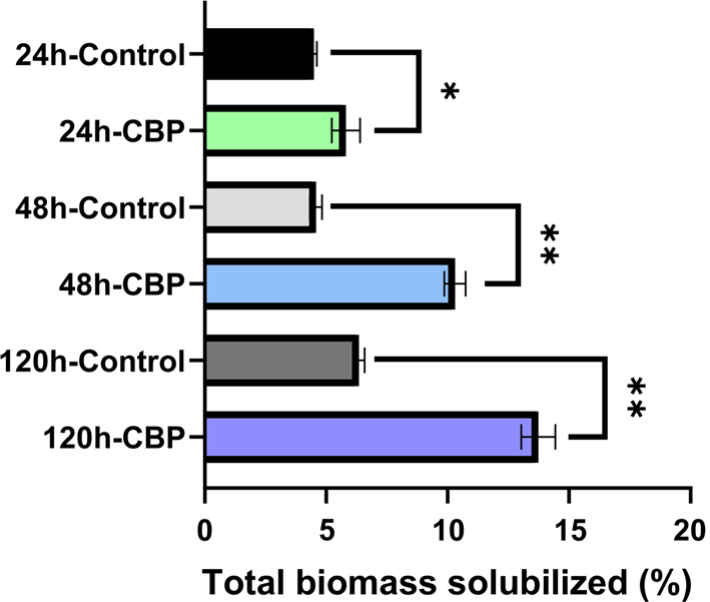
Total solids solubilization of CBI reference poplar biomass by *C. thermocellum* DSM1313 over 5 days at 60 °C. Poplar biomass was loaded at 5.68 g per 500-mL reaction volume at the start of fermentation to achieve a 5.0 g/L glucan loading at 0.5-L bioreactor scale. Uninoculated controls were included to account for any non-biological solubilization. The percentage of total biomass solubilization was calculated from the data presented in Additional file 2: Fig. S2. Data are average of two technical replicates from each of two biological replicates (i.e., two separate fermentations) ± standard deviation, *n* = 4. Statistical analysis was by one-way ANOVA followed by Fisher’s least significant difference method; *P < 0.05, **P < 0.01

A schematic of the microbial fermentation and analysis procedure used to evaluate the solubilization of poplar biomass by *C. thermocellum* DSM1313 is shown in Additional file 1: Fig. S1. Fermentations (0.5 L) were performed with 5.68 g chipped and milled poplar biomass at 5 g/L total glucan loading corresponding to approximately 11.4 g/L total solids loading. Changes in total mass yield and cellulosic and non-cellulosic sugar composition in the liquid and solid fractions were measured at times zero, 24, 48 and 120 h (5 days). Microbial fermentation in pH-controlled reactors inoculated with *C. thermocellum* resulted in total solids solubilization of 5.8%, 10.3%, and 13.7% after 24, 48, and 120 h, respectively, while control reactors without the microbe (henceforth called fermentation controls) resulted in 4.5%, 4.6% and 6.3% total solids solubilization at the same time points (Fig. 1; Additional file 2: Fig. S2). The total amount of biomass solubilized specifically by *C. thermocellum* CBP was calculated by subtracting the end-point biomass weight recovered after CBP fermentation from the starting biomass, as described in the material and methods, and subtracting from this the amount of biomass solubilized by the fermentation controls which did not contain the microbe. When the amount of biomass solubilized due to the heat and medium conditions in the reactors (i.e., as in the fermentation controls) was subtracted, the results showed that *C. thermocellum* CBP solubilized 1.3%, 5.7% and 7.4% of the poplar biomass after 24, 48, and 120 h, respectively.

### Evaluation of dry mass content and glycosyl residue composition of post-fermentation liquor from *C. thermocellum* CBP fermentations

**Fig. 2:**
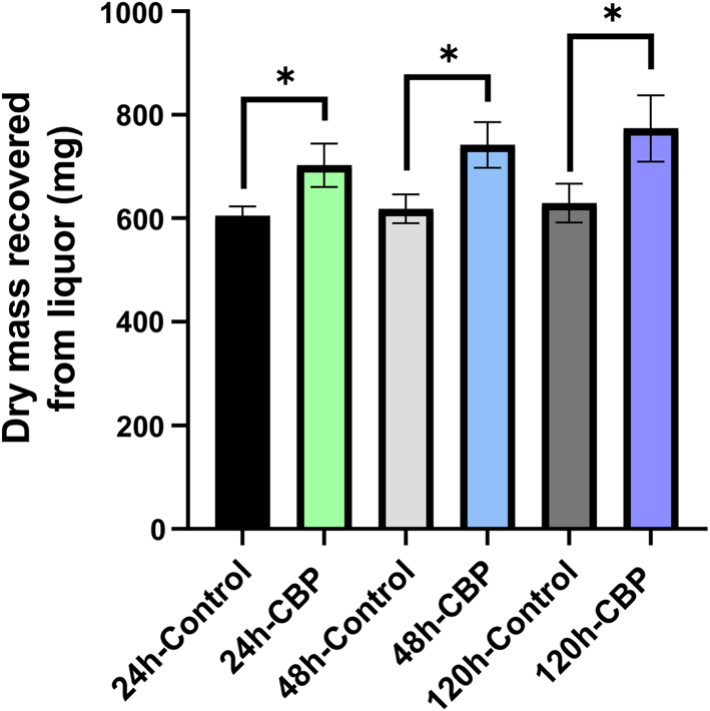
Total dry mass recovered from the fermentation liquor after solubilization of milled un-pretreated CBI reference poplar biomass by *C. thermocellum* DSM1313 over 5 days at 60 °C. Dry mass recovered from uninoculated controls was included to account for any non-biological solubilization. Liquor dry mass was collected post-fermentation as described in Additional file 1: Fig. S1. Data are average from two technical replicates of each of two biological replicates (i.e., two fermentations) ± standard deviation (SD), *n* = 4. Statistical analysis was with one-way ANOVA followed by Fisher’s least significant difference method; *P < 0.05

After 24, 48, and 120 h of microbial and control fermentations, the content of each fermentation reactor was centrifuged to separate the liquor (i.e., the supernatant representing the soluble fraction) from the solid residue. The CBP and fermentation control liquor samples were lyophilized for five days and the dry mass was recorded. There was a significant increase (16–23%) in the dry mass recovered from the CBP liquor compared to the controls at 24, 48, and 120 h, respectively (Fig. 2). These results showed that *C. thermocellum* solubilized biomass over the course of the fermentations. Since the amount of dry mass recovered from the liquor of the fermentation controls and the CBP fermentations was greater than the amount of total solids solubilized from the biomass (Fig. 2; Additional file 2: Fig. S2), these results suggested that the additional mass recovered in the control liquor may have resulted from dried culture medium components and that the additional mass recovered in the *C. thermocellum* fermentations may have resulted from the culture medium components plus microbe culture components.

**Fig. 3:**
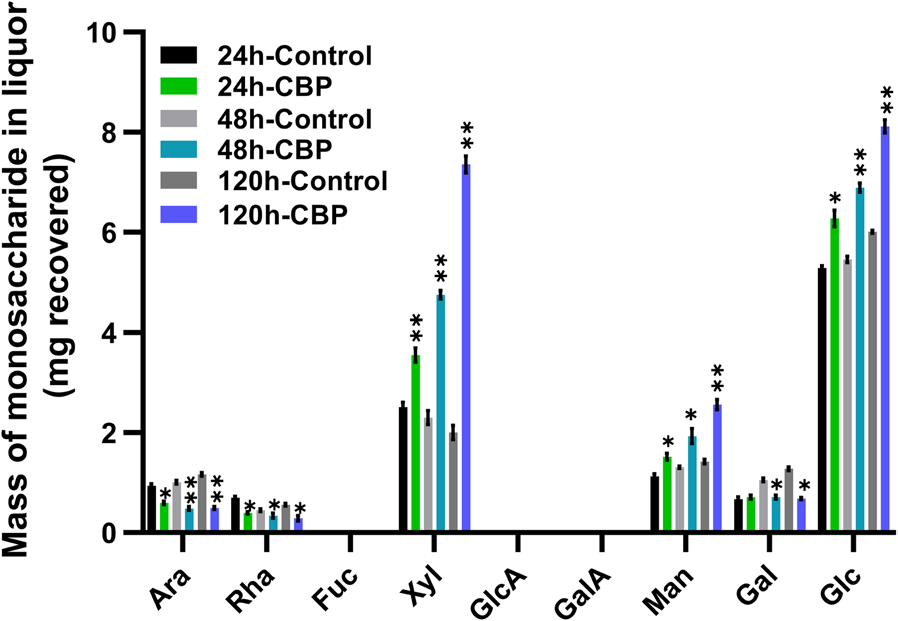
Glycosyl residue composition of post-fermentation liquor as described in Fig. 2 by trimethylsilyl (TMS) derivatization and GC–MS. The amount of sugar is represented as average mg recovered in the designated liquor from 5.68 g of starting biomass. Mean ± SD of two biological (i.e., two fermentations) and two technical replicates (*n* = 4). P values are *P ≤ 0.05, **P ≤ 0.001 at significant level (one-way ANOVA followed by Fisher’s least significant difference method)

In order to determine which components of the poplar biomass were solubilized by *C. thermocellum* and recovered in the liquor during the CBP fermentations, the liquor samples from the control and *C. thermocellum* fermentations were analyzed for glycosyl residue composition by trimethylsilyl (TMS) derivatization and GC–MS. This method specifically quantifies the non-cellulosic sugar composition [17]. Notably, large amounts of non-cellulosic sugars were present in the fermentation controls, indicating that some of the non-cellulosic polysaccharides in the poplar biomass were solubilized due to the temperature setting and medium used during the fermentation. Significantly more sugars, however, were solubilized from the poplar biomass by *C. thermocellum* fermentations compared to the controls including 41–267% more xylose (Xyl), 35–80% more mannose (Man), and 19% to 35% more glucose (Glc) over the 120 h fermentation (Fig. 3 and Additional file 3: Fig. S3). The accumulation of these sugars in the liquor suggested that *C. thermocellum* was able to solubilize hemicelluloses, such as glucuronoxylan and glucomannan in poplar wood, but did not completely take up or metabolize these sugars. Only trace amounts of acidic sugars (e.g., galacturonic acid, GalA) were detected in the liquid fractions.

Interestingly, arabinose (Ara), rhamnose (Rha), and galactose (Gal) were also detected in the liquor of both the control and *C. thermocellum* fermentations. However, their levels were significantly less in the liquor from the CBP fermentations compared to the liquor from the fermentation controls, being 36–57% less for Ara, 24–49% less for Rha, and 32–46% less for Gal at 48 h and 120 h). The reduction in the levels of these sugars in the liquor of the *C. thermocellum* fermentations compared to the control fermentations, suggested that *C. thermocellum* assimilated these sugars. Since the levels of these sugars were greater in the control liquor than in the CPB liquor, it was not possible from these data alone to conclude whether or not *C. thermocellum* had solubilized these sugars from the biomass, or rather they had all resulted from the process conditions. To determine how much sugar *C. thermocellum* was able to solubilize from the biomass, the solid residues obtained during the fermentations were also evaluated.

### Production of alcohol-insoluble residues from poplar residual biomass and characterization of lignin, cellulose, and non-cellulosic sugars in the residual biomass

In order to determine the amount of lignin, cellulose and non-cellulosic polysaccharides remaining in fermentation residues during *C. thermocellum* CBP, alcohol-insoluble residue (AIR) was produced from the post-fermentation biomass (solid residues) remaining after CBP and in the fermentation controls. The resulting AIR, which represents cell walls, was de-starched for 48 h with α-amylase and the resulting de-starched AIR was analyzed for cellulose, lignin and non-cellulosic sugars. The yields of total AIR per gram of solid residue from the fermentation controls and the *C. thermocellum* CBP fermentations after the first 24 h were comparable (Additional file 4: Fig. S4). However, the yields of AIR per gram of solid residue from the CBP solid residuals were 3% and 5% less than that from the controls after 48 and 120 h incubation, respectively. This slightly lower yield of AIR per gram solid residue in the *C. thermocellum* CBP fermentations may have been due to components contributed to the solid residues by *C. thermocellum* over the course of the fermentation that were not solubilized by the ethanol and chloroform/methanol extraction process used for AIR preparation.

#### Lignin content

**Fig. 4:**
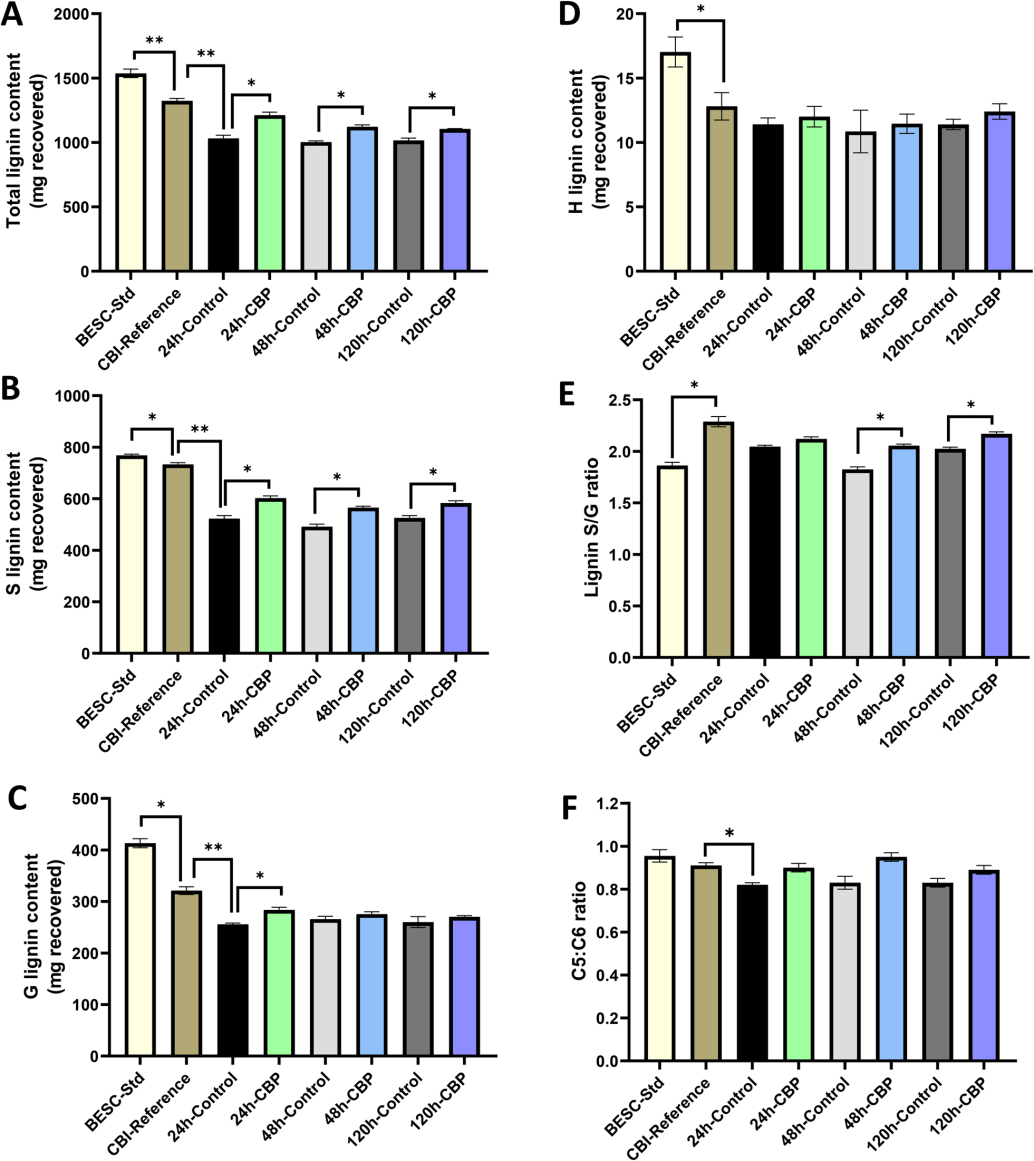
Lignin content and S/G ratio of the starting biomass and of the solid residues recovered after *C. thermocellum* CBP bioconversion. **A** Total lignin, **B** S lignin, **C** G lignin, **D** H lignin content, and **E** S/G ratio and **F** C5:C6 ratio. Data are mg recovered from the starting biomass or mg recovered from the solid residues from poplar biomass after CBP bioconversion with *C. thermocellum* over 120 h compared to fermentation controls. Statistical analysis was by one-way analysis of variance (ANOVA) followed by Fisher’s method. Mean ± SD, significant P values are expressed as *P < 0.05, **P < 0.01

To evaluate the amount and composition of lignin in the post-fermentation solid residues of poplar wood, the amount of total lignin, guaiacyl (G), *p*-hydroxyphenyl (H) and syringyl (S) lignin subunits and the lignin S/G ratio were measured by pyrolysis molecular beam mass spectrometry (py-MBMS) [4]. The total lignin content of the CBI reference poplar was significantly less (14%) than the total lignin content of the BESC standard poplar (Fig. 4A; Additional file 5: Fig. S5A). The CBI reference poplar line (GW-9947) was previously identified as a low recalcitrance poplar natural variant with reduced lignin and a high syringyl-to-guaiacyl (S/G) ratio [18], a difference confirmed by the py-MBMS lignin analysis results reported here.

Analysis of the lignin content of the CBI reference poplar biomass versus the lignin content in the solid residues of the 24 h, 48 h, and 120 h CBP fermentation controls revealed an unexpected reduction in the amount of total, S, and G lignin, suggesting that some lignin-containing wall material is solubilized during the process used for fermentation. The percentage of total, S, and G lignin content of the recovered CBP solid residues following fermentation of the CBI reference poplar by *C. thermocellum* increased on average 25%, 24%, and 13%, respectively, compared to the fermentation controls (Fig. 4A–D; Additional file 5: Fig. S5), whereas no major changes were observed in the H subunit content. The increase in the percentage of lignin in the residual biomass during CBP fermentation by *C. thermocellum* was at least partially associated with the solubilization of hemicellulose and cellulose (see below)*,* leading to a greater weight percentage of total lignin in the residues.

The lignin S/G ratios were significantly higher (6–14%) in CBP solid residue compared to those of controls (Fig. 4E). A higher S/G ratio in the residual solids following *C. thermocellum* CBP fermentation of poplar wood has been previously described [16, 18–20]. One hypothesis for explaining this result is that some of the hemicellulose or cellulose solubilized by *C. thermocellum* is associated with lignin that has a higher G/S ratio, which was preferentially solubilized along with the glycans. There were no major changes in the C5:C6 ratio of poplar solid residues in the control or *C. thermocellum* CBP fermentations (Fig. 4F).

#### Cellulose content

**Fig. 5:**
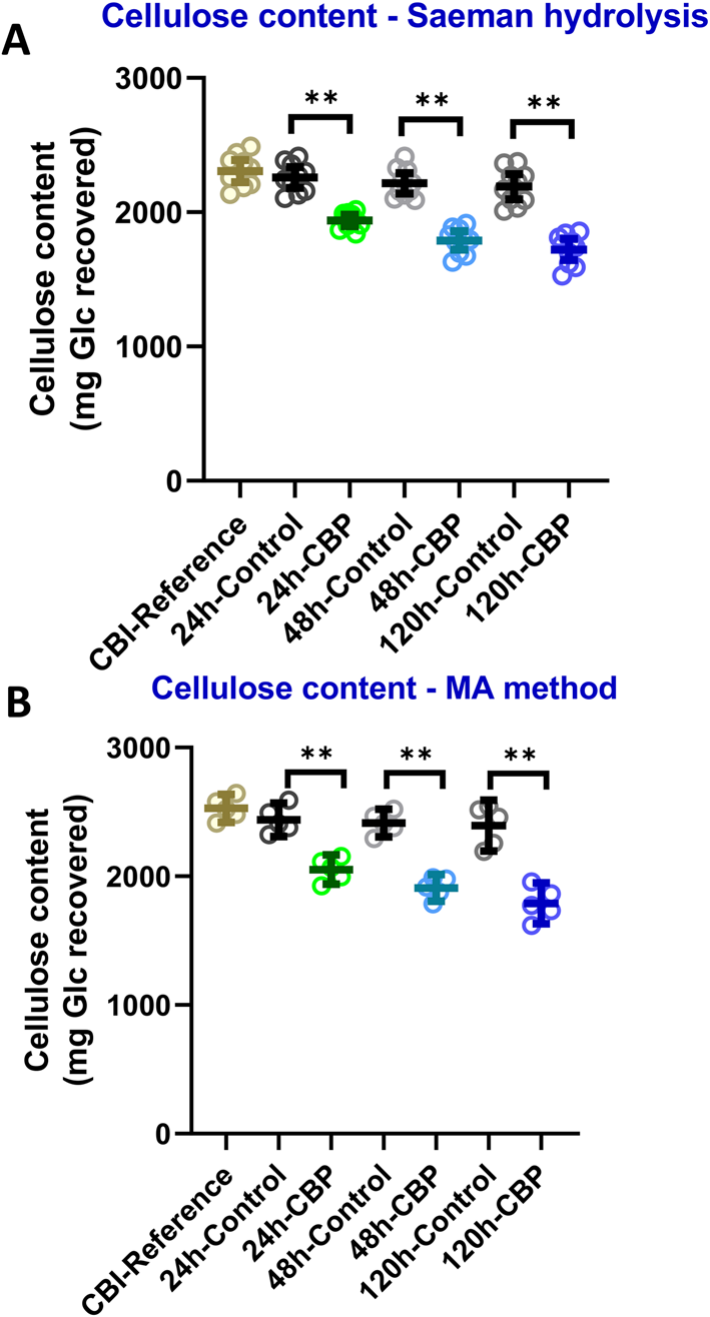
Cellulose content of the starting biomass and of the solid residues recovered during *C. thermocellum* fermentation and in fermentation controls. Cellulose content of alcohol-insoluble residue (AIR) (i.e., cell walls) of solid residue recovered after consolidated bioprocessing and in fermentation controls by **A** Saeman hydrolysis [21, 22] and **B** methylated alditol method [25]. See Methods for details. The amounts of sugar are represented as average mg of glucose recovered from the starting biomass (5.68 g). Mean ± SD of 2 biological and three technical replicates (*n* = 6). *P* value is **P ≤ 0.01 at significant level

Two methods were used to determine the cellulose content in the post-fermentation solid residues. The first method involved measuring the amount of glucose present in non-crystalline polysaccharides (i.e., non-cellulosic cell wall glycans) by GC–MS of TMS-derivatized methyl glycosides and subtracting this value from the total cellulosic plus non-cellulosic glucose as obtained by Saeman (1945) hydrolysis of cell wall material [21, 22] followed by glycosyl residue composition analysis (Additional file 6: Fig. S6A–C). This measurement of cellulose (Fig. 5A; Additional file 6: Fig. S6C) was in good agreement with published values of cellulose content for poplar wood [23, 24]. The second method used to measure cellulose content was the recently developed fully methylated alditol (MA) procedure which enables analysis of insoluble polysaccharides through their permethylation in DMSO to produce methyl alditol derivatives that can be analyzed by GC–MS [25] (Fig. 5B; Additional file 6: Fig. S6D–F). The MA method thus also provides a measure of total Glc which, upon subtracting the amount of non-cellulosic Glc (see subsection below), provides the cellulose content. CBP fermentation of CBI reference poplar biomass for 5 days by *C. thermocellum* resulted in a significant 25% total reduction in the cellulose content (mg Glc/starting biomass) based on the Saeman hydrolysis method, which corresponded to a 21% reduction after subtracting the fermentation controls (Fig. 5A; Additional file 6: Fig. S6A–C). The MA method indicated a 29% total reduction in cellulose (mg Glc/starting biomass) and 25% reduction after subtracting the fermentation controls (Fig. 5B; Additional file 6: Fig. S6D–F; Table 1). These values are comparable to those of Dumitrache and coworkers [23] who showed a 28% reduction in total glucose content of *P. deltoides* biomass after incubation of *C. thermocellum* ATCC27405 for 92 h at 60 °C and confirmed that the microbe can solubilize a portion of the cellulose in non-pretreated poplar woody biomass.

**Table 1:** Mass of lignin, cellulosic and non-cellulosic sugars in starting biomass and residual solids during CBP by *C. thermocellum*

Cell wall component	Starting biomass (mg)^a^	Final solid residue (mg)		Amount solubilized by *C. thermocellum* CBP (mg)		Percentage (%) solubilization by C. *thermocellum* CBP		
		Control	CBP	Gross	Net	Gross	Net	Net^b^
Cellulosic sugar								
Glc (SH)	2307	2191	1722	585	469	25	20	21
Glc (MA)	2528	2394	1789	739	605	29	24	25
Non-cellulosic sugar								
Ara	54	49	42	12	7	23	13	15
Rha	25	20	25	**0**	**− 5**	**0**	**− 21**	**− 27**
Fuc	5	4	3	2	1	32	12	15
Xyl	1249	1160	995	253	165	20	13	14
GlcA	13	12	12	1	**0**	11	3	3
GalA	107	83	24	82	58	77	55	71
Man	65	61	39	26	22	40	34	36
Gal	53	49	42	11	7	21	12	13
Glc	158	148	114	44	34	28	21	23
Total	1729	1584	1296	433	288	25	17	18
Lignin								
S lignin	733	525	584	149	**− 59**	20	**− 8**	**− 11**
G lignin	321	260	270	51	**− 10**	16	**− 3**	**− 4**
H lignin	13	11	12	1	**− 1**	8	**− 8**	**− 9**
Total lignin	1323	1017	1105	218	**− 88**	16	**− 7**	**− 9**

All data are mg per fermentation reaction, rounded to nearest integer. Zero and negative values are in bold. Gross—total solubilization of residual pellet in CPB fermenter. Net—solubilization of CPB fermenter residual pellet minus fermentation control residual pellet. Percentage solubilization is mass % of sugar in starting biomass

*SH* saeman hydrolysis, *MA* methylated alditol

^a^The amount of starting biomass was 5.68 g

^b^Net percent solubilization is mass % of net sugar in final residue of fermentation control

#### Glycosyl residue composition of non-cellulosic sugars in solid residues

**Fig. 6:**
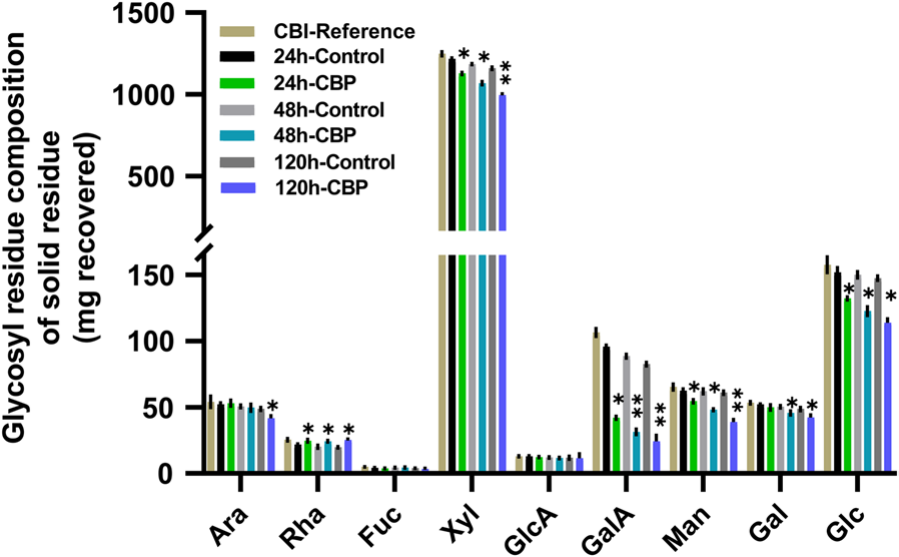
Glycosyl residue composition by trimethylsilyl (TMS) derivatization and GC–MS of AIR (alcohol-insoluble residues) from 5.68 g starting biomass and recovered from the solid residues after fermentation of CBI reference poplar biomass by *C. thermocellum*. Mean ± SD of two biological and two technical replicates (*n* = 4). Stars indicate values that are different at *P ≤ 0.05, **P ≤ 0.001 significant level

To study the solubilization of non-cellulosic cell wall glycans by *C. thermocellum* CBP, the glycosyl residue composition of CBI reference poplar solid residues after 24, 48, and 120 h of microbial hydrolysis was measured and compared to those of the solid residues in the fermentation controls (Fig. 6). Glycosyl residue composition analysis of the solid residues before and after *C. thermocellum* CBP revealed that the mg per mass starting biomass of six sugars was significantly decreased in the presence of *C. thermocellum* (Fig. 6) with 7–14% decreased Xyl, 13–36% decreased Man, 13–23% decreased Glc, and 56–71% decreased GalA from 24 to 120 h. There was also 9–13% decreased Gal at 48 h to 120 h and 15% decreased Ara at 120 h indicating hydrolysis by *C. thermocellum*. Similar relative changes in post-fermentation glucose content (18% decreased Glc/gram solid) has been reported for other high S/G woody phenotypes [19]. The particularly large reduction in the amount of GalA in the solid residue but little GalA in the liquor suggests the possible utilization of pectic sugars by *C. thermocellum*. Conversely, there was a significant trend for increased Rha (12–21%) in the CBP solid residues compared to the fermentation controls, suggesting that Rha-containing polymers in the wall (e.g., RG-I) were not being solubilized and thus were found to proportionally increase in the final residue recovered. Although there was a small reduction in the total non-cellulosic sugars in the solid residue from the fermentation control, the 10% to 18% greater reduction in total non-cellulosic sugars in the solid residues following CBP fermentation by *C. thermocellum* indicates that the microbe solubilizes at least some of the non-cellulosic wall polymers including xylans and homogalacturonan (Fig. 6; Additional file 7: Fig. S7).

**Fig. 7:**
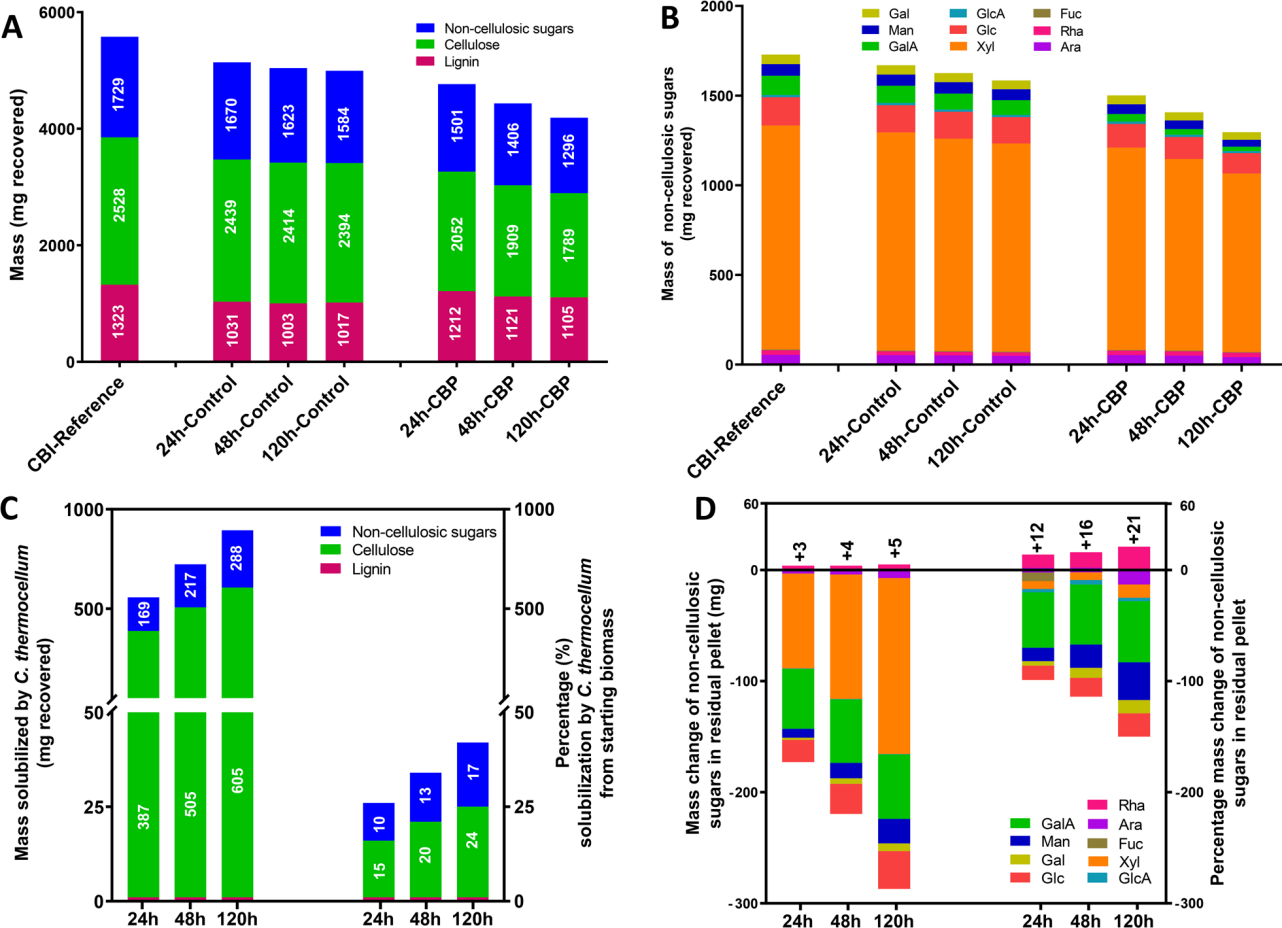
Total mass and % solubilization of lignin, cellulose, and non-cellulosic sugars from solid residues remaining after CBP fermentation of 5.68 g starting milled poplar biomass. Data are mg recovered from the starting biomass, mg recovered from the solid residues, or % solubilized, as indicated. **A** Mass (mg recovered) of total lignin, cellulose, and non-cellulosic sugars in the solid residues. These data are from Figs. 4, 5 and 6. **B** Mass (mg recovered) of total non-cellulosic sugars (Xyl, Glc, Man, Ara, Gal, Rha, Fuc, GalA, GlcA) in the solid residues at the times indicated. The mass of total monosaccharide is from data from Fig. 6. **C** Mass (left) and percentage (right) of non-cellulosic sugars, cellulose and lignin solubilized by *C. thermocellum*. All solubilization calculations were done based on the starting biomass. **D** Mass change and percentage of mass change of net non-cellulosic sugars solubilized by *C. thermocellum*. Both mass and percentage values are calculated as described in Table 1. The 24 h and 48 h % net solubilization values from starting biomass are 24 h: Glc (13%), Gal (4%), Man (12%), GalA (50%), GlcA (3%), Xyl (7%), Fuc (10%), Rha (− 12%), Ara (− 2%). 48 h: Glc (17%), Gal (9%), Man (21%), GalA (54%), GlcA (4%), Xyl (9%), Fuc (0%), Rha (− 16%), Ara (2%)

A comparison of the amount of lignin, cellulose, and non-cellulosic sugars in the solid residues recovered over the 120 h fermentation (Fig. 7) shows that 24% percent of the cellulose was solubilized over 5 days of CBP fermentation by *C. thermocellum* (Fig. 7A, C). Simultaneously, 17% of the non-cellulosic sugars were solubilized suggesting that *C. thermocellum* hydrolyzes these sugars during fermentation (Fig. 7B). The negative value of mass and percentage of lignin solubilization in Table 1 is due to the increase in the proportional lignin content in the residual biomass as the cellulose and non-cellulosic sugars are solubilized, resulting in no evidence for solubilization of lignin by *C. thermocellum* (Fig. 7C; Table 1). An analysis of the percentage of the specific sugars solubilized (Fig. 7D; Table 1) revealed that Rha was the only sugar that was not solubilized by *C. thermocellum* during the fermentation. This result suggested that Rha-containing cell wall polymers (i.e., RG-I) may be a key determinant limiting solubilization of poplar biomass during CBP fermentation by *C. thermocellum*.

**Fig. 8:**
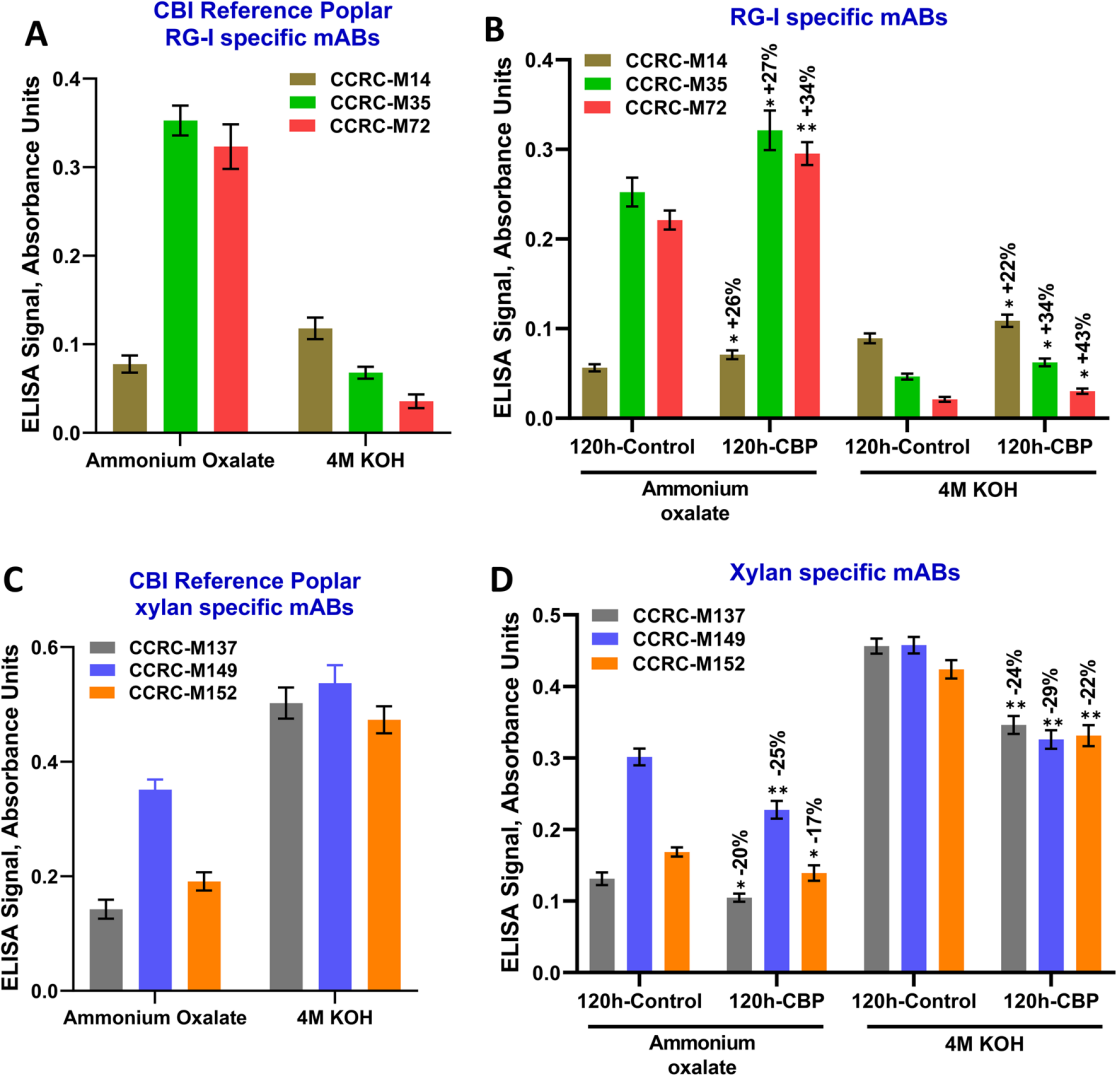
ELISA absorbance signals from CBI reference poplar and solid residues recovered after CBP fermentation. Sequential extracts of cell walls from solid resides were prepared and used for the ELISA assay. ELISA signals from RG-I backbone specific mAbs CCRC-M14, CCRC-M35, and CCRC-M72 [26, 27] on **A** CBI reference poplar and **B** solid residues recovered after 120 h fermentation by *C. thermocellum*. ELISA signals from xylan-specific mAbs CCRC-M137, CCRC-M149, CCRC-M152 [26–28] on **C** CBI reference poplar and **D** solid residues recovered after 120 h CBP fermentation. Mean ± SD of two biological and four technical replicates (*n* = 8). Stars indicate values that are different at *P ≤ 0.05, **P ≤ 0.001 significant level

To test this, monoclonal antibodies reactive against the RG-I backbone were used in enzyme-linked immunosorbent assays (ELISA) to measure the relative amount of RG-I in the starting biomass and in the solid residues of the 120-h *C. thermocellum* fermentation and 120-h fermentation control. Since the solid residues could not be bound to the ELISA plate, they were sequentially extracted with 50 mM ammonium oxalate and 4 M KOH, solvents known extract non-cellulosic polysaccharides [4]. Based on the relative increase in the amount of Rha in the solid residues from the *C. thermocelllum* fermentation (Figs. 6, 7), we hypothesized that the relative RG-I signal detected in the solid residues of the CBP 120-h fermentation would be greater than that detected in the fermentation control. To test this hypothesis, three antibodies directed against the backbone of RG-I (CCRC-M14, CCRC-M35, CCRC-M72) were used [26, 27]. These antibodies had strong signals with the ammonium oxalate and 4 M KOH extracts of the CBI reference poplar (Fig. 8A), confirming the presence of RG-I in poplar biomass [4]. As shown in Fig. 8B, the ELISA signals for all three RG-I specific antibodies were significantly increased in the ammonium oxalate (26–34%) and 4 M KOH (22–43%) extracts of CBP solid residues compared to the fermentation controls. Glycosyl residue composition analysis revealed that xylose decreased significantly in the solid resides recovered after CBP compared to the fermentation controls. Therefore, as a further control xylan-specific antibodies directed against the β-1,4-linked xylan backbone (CCRC-M149), as well as anti-xylan antibodies that could detect unsubstituted and partially arabinose-substituted xylan backbone (CCRC-M137, CCRC-M152) [26–28] were also used. Strong signals were obtained in ELISA analysis of ammonium oxalate and 4 M KOH extracts of CBI reference poplar (Fig. 8C). Furthermore, as expected, all three xylan-specific antibodies had significantly decreased ELISA signals in the ammonium oxalate (17–25%) and 4 M KOH (22–29%) extracts of the CBP solid residues compared to the fermentation controls (Fig. 8D). These results confirmed the glycosyl residue composition results (Fig. 6) and showed that while *C. thermocellum* effectively solubilized xylan, it was not able to effectively solubilize the RG-I in the poplar biomass.

## Discussion

*C. thermocellum* secretes an extensive array of glycoside hydrolases with more than 70 types of cellulosomal proteins capable of hydrolyzing different types of lignocellulosic biomass [29–32]. However, without pretreatment of the lignocellulosic biomass, this cellulolytic microbe cannot completely depolymerize the native complex polysaccharides in the plant cell wall of either woody and mature grassy feedstocks. The objective of this study was to characterize the solubilization of both cellulose and non-cellulosic sugars from non-pretreated milled CBI reference poplar biomass [18] by *C. thermocellum* by evaluating the starting biomass, the post-fermentation liquors, and solid residues recovered during the CBP process. Our goal was to quantify the amount of cellulosic and non-cellulosic sugars solubilized during the CBP process to provide information about which polymers in woody biomass *C. thermocellum* solubilizes and to identify potential limiting factors that could inform the development of an economically viable consolidated bioprocess.

*C. thermocellum* can efficiently solubilize crystalline cellulose, with reports of 95% and 93% solubilization of Avicel™ at low (5 g L^−1^) [33] and high (100 g L^−1^) [34] solids loadings, respectively. However, it has been reported to achieve a much lower 17% solubilization of woody *Populus* biomass at low (10 g L^−1^ dry weight, DW) solids loading and the solubilization was affected by the composition of the biomass with 12% solubilization of low S/G lignin poplar (20 g L^−1^ DW) and 20% solubilization of high S/G lignin poplar (20 g L^−1^ DW) [19]. The amount of solubilization of biomass by *C. thermocellum* is affected by biomass loading, with total cellulose solubilization decreasing from 76 to 58% and 63% to 37% as switchgrass loadings were increased from 9.2 to 92 g L^−1^ and 10 to 50 g L^−1^ (dry weight), respectively, although Avicel™ was completely consumed at all loadings [35, 36]. In the work reported here, we achieved 14% solubilization of poplar wood at 11.4 g L^−1^ solids loading after 5 days of fermentation (Fig. 1; Additional file 1: Fig. S1). Earlier results and those reported here confirm that *C. thermocellum* can solubilize non-pretreated woody lignocellulosic biomass, but that additional pretreatment or alternative biomass modifications are necessary to achieve efficient conversion of biomass at high solids loadings [35, 37].

The plant biomass-solubilizing enzymes produced by C. *thermocellum* are expressed and mostly arranged on multi-enzyme structures known as cellulosomes and it appears that the microbe can regulate the enzymes on the cellulosomes based on the substrate and growth conditions [29–32, 38]**.** *C. thermocellum* hydrolysis of cellulose in poplar biomass was reported to result in solubilization of 17–18% of the glucan in recovered post-fermentation solid residues compared to the controls [19]. Another study, using confocal microscopy, revealed a 49% reduction of cellulose signals in post-fermentation biomass and a concomitant 28% reduction in total glucose after CBP with *C. thermocellum* on *P. deltoides* [23]. In the current study, the cellulose content in post-fermented CBP solid residues was shown to decrease 21% and 25% based on Saeman hydrolysis (SH) and methylated alditol (MA) analysis, respectively (Fig. 5). These results compare well with the prior studies [19, 23]. The limited hydrolysis by C. *thermocellum* of cellulose in poplar biomass results in 75–79% of the cellulose being retained in the poplar solid residues after 5 days of fermentation.

It is interesting to consider the effect of lignin quantity and composition on the ability of *C. thermocellum* CBP to solubilize cellulose in poplar biomass. The CBI reference poplar has a mutation in the 5-enolpyruvylshikimate-2-phosphate (EPSP) synthase gene and is among multiple low recalcitrance poplar natural variants affected in lignin biosynthesis with high S/G ratios that have been positively correlated with glucan solubilization [18]. Here, we confirmed that the total lignin content of the CBI reference poplar (23.3%) is significantly lower than that of the BESC standard poplar (27.1%) on a biomass dry weight basis [18]. Analysis of the fate of the lignin in the post-fermentation solid residues of the CBI reference poplar showed that after CBP there was greater total lignin (25%), syringyl (S) lignin (22–26%), and guaiacyl (G) lignin (11–15%) content and a higher S/G ratio (6–14%) in the solid residues, but no major change in the *p*-hydroxyphenyl (H) subunits compared to the fermentation control solid residues (Fig. 4A–E; Additional file 5: Fig. S5). These results are consistent with prior reports indicating that S/G ratios and lignin composition impact hydrolysis of poplar wood by *C. thermocellum* and that relative lignin content increases in the insoluble residue generated during CBP fermentation [18, 19, 23]. The substantial relative increase in the S- and G-lignin in the CBP fermentation solid residues agrees with the earlier finding that lignin accessible surface increased (S-lignin increased 30%, G-lignin increased 11%), whereas the cellulose surface decreased (49%) during microbial hydrolysis in poplar [23].

The hemicellulose content in poplar species is reported to range from 16 to 23% [24, 39]. The main non-cellulosic polysaccharide in hardwoods is the hemicellulose glucuronoxylan. Xylan constitutes from 13.4% to 22.4% of wood dry weight in poplar [24, 39]. The second most abundant non-cellulosic polysaccharide in hardwoods is either glucomannan or pectin [39]. In addition to cellulases, cellulosomes from *C. thermocellum* contain xylanases, pectinases, and other accessory glycoside hydrolase enzymes [29, 30]. For example, *C. thermocellum* expresses multiple hemicellulose degrading enzymes, including xylanases (XynA, XynC, XynD, XynE, XynY, XynZ) [40–45], xyloglucanase (XghA) [42], mannanase (Man26A, B) [46, 47], and other hemicellulases, such as (β-1,3-1,4-glucanase, LicB [48] and a chitinase (endochitinase, Chi18A [49]) the latter which, although not a hemicellulase hydrolyzes a structure with a comparable β-1,4-linked backbone. Interestingly, all the known cellulosomal xylanases contain either the carbohydrate-binding domain 22 (CBM22) or CBM6 which bind to xylan and cellulose [43, 50], suggesting that these enzymes are specifically targeting xylan associated with cellulose. However, although *C. thermocellum* is able to break down xylan, it is unable to ferment hemicellulose solubilized from the lignocellulosic biomass [29, 51]. Accordingly, we show that the relative amounts of non-cellulosic sugars attributable to hemicelluloses present in the liquor during *C. thermocellum* CBP fermentation are significantly greater than the amounts in the liquor of the fermentation controls. Specifically, the liquor during *C. thermocellum* CBP fermentation contained 41–267% more xylose (69–368% mg/total solubilized liquor); 35% to 80% more mannose (61–130% mg/total solubilized liquor); and 19–35% more non-cellulosic glucose (42–72% mg/total solubilized liquor) (Fig. 3; Additional file 3: Fig. S3) per mg/starting biomass than the liquor of the fermentation controls over the 120 h incubation period. Concomitantly, there was a significant 10–18% reduction in the total non-cellulosic sugar content in the post-fermentation CBP solid residues from 24 to 120 h (Fig. 6; Additional file 7: Fig. S7) including a 7–14% mg/starting material reduction in xylose, a 13–36% mg/starting material reduction in mannose and a 13–23% mg/starting material reduction in non-cellulosic glucose. The retention of significant amounts of the solubilized Xyl, Man, and non-cellulosic Glc in the fermentation liquor supports the hypothesis that *C. thermocellum* cannot grow on pentose sugars and also is not able to ferment hemicellulosic sugars like xylose and mannose from poplar wood [29, 52]. The hemicellulose-solubilizing enzymes are suggested to function by removing hemicelluloses from the biomass in order to expose the preferred cellulose substrate to *C. thermocellum* for effective cellulosomal solubilization [29, 53].

Enzymatic and chemical removal of xylan is known to enhance the accessibility of glucanases to cellulose in lignocellulosic biomass [54]. However, in the current study, the total solids solubilization of biomass by CBP was only 14% greater than in the fermentation controls. It has previously been reported that xylose oligomers have inhibitory effects on the enzymatic hydrolysis, conversion rates, and hydrolysis yield of *C. thermocellum* grown on plant biomass, as well as the hydrolysis of Avicel™ [35, 55–57]. When considered together with the reduced relative xylose content in the solid residues (Fig. 6; Additional file 7: Fig. S7), the increased relative amounts of xylose in the *C. thermocellum* CBP liquor (Fig. 3; Additional file 3: Fig. S3) are consistent with the hypothesis that xylose oligomers are accumulating in the liquor and may be inhibiting the solubilization of poplar wood by *C. thermocellum*. These results indicate that efforts to enable *C. thermocellum* to metabolize and utilize hemicellulose may enable better solubilization of poplar biomass.

*C. thermocellum* expresses multiple pectin solubilizing enzymes including three endopectin lyases (PL1A, PL1B and PL9) [58] and a reported rhamnogalacturonan I (RG-I) lyase (PL11 family: RGLf [59]). RGLf has activity against homogalacturonan and RG-I preparations containing homogalacturonan. All these enzymes exist as modular enzymes that contain a carbohydrate binding module and function in cellulosomes. Interestingly, whereas PL1A contains the carbohydrate-binding domain 6 (CBM6) that binds to cellulose or hemicellulose, RGLf, as well as the multifunctional PL1B and PL9, contains CBM35 that has recently been shown to bind RG-I [60], indicating that solubilization of pectin is genetically programmed into *C. thermocellum* for biomass solubilization. The cell wall sugar composition analyses reported here show the largest reduction of GalA (56–71%) content compared to the other non-cellulosic sugars in the CBP biomass residues compared to the fermentation controls (Fig. 6; Additional file 7: Fig. S7; Table 1). These results suggest that *C. thermocellum* efficiently utilizes polygalacturonic acid (homogalacturonan) in poplar wood as a carbon source, as previously reported for sugar beet [15]. Surprisingly; however, there was a significant increase in the relative amounts of rhamnose (Rha, 12–21%), a major sugar in the RG-I backbone, in the solids residue after the first 24 h of *C. thermocellum* fermentation compared to the control fermentations, suggesting that *C. thermocellum* was not efficiently solubilizing RG-I. This was confirmed by ELISA analysis of the solid residues identifying an increased relative amount of RG-I in the solid residues following CBP fermentation. There was no change in the amount of arabinose and galactose, sugars abundant in the side branches of RG-I.

Compared to the fermentation control, *C. thermocellum* hydrolysis of poplar biomass removed only 17% more of the total non-cellulosic sugars after 5 days of fermentation, indicating that the residual poplar wood still retained the bulk of the non-cellulosic wall polysaccharides (Additional file 7: Fig. S7B). Because fermentation of non-pretreated poplar by *C. thermocellum* resulted in 7% total solids solubilization compared to the fermentation control (Fig. 1) and there was a greater retention of glycosyl residues associated with RG-I in the post-fermentation solid residues as well as an increased ELISA signal for RG-I backbone epitopes compared to the fermentation control, we hypothesize that RG-I and its side chains may be resistant to solubilization by *C. thermocellum* and potentially contribute to the limited hydrolysis of non-pretreated woody biomass by *C. thermocellum*. Our current hypothesis is supported by the recent finding that poplar wood RG-I is a key determinant of cell–cell adhesion in wood [11]. Yang and coworkers showed that treatment of poplar wood with acidic chlorite and dilute alkali after digestion with the RG-I-degrading enzyme RG-lyase resulted in more separation of the poplar wood into single cells than treatment with acidic chlorite and dilute alkali alone, supporting a role for RG-I in cell–cell adhesion. Cell separation in the woody biomass was also increased by reducing the RG-I content in the wood by transgenic expression of an *Arabidopsis thaliana* gene encoding an RG-lyase (*AtRGIL6*) in poplar [11], confirming a role for RG-I in wood cell wall architecture and cell–cell adhesion.

The cellulosomal cellulolytic activities and non-cellulosic xylanases and pectinolytic activities makes *C. thermocellum* a promising CBP microbe for production of fuels and chemicals from lignocellulosic biomass. The microbe by itself; however, is not able to achieve effective solubilization of all the cellulosic and non-cellulosic polysaccharides from some biomass feedstocks. The low percentage solubilization of the untreated woody biomass is a bottleneck. We previously reported that pectin contributes to biomass recalcitrance and saccharification yield although it is present in relatively low abundance in woody biomass [4, 61–63]. Furthermore, the deletion of a pectinase gene cluster in the thermophile Caldicellulosiruptor* bescii* resulted in reduced growth of the mutated microbe on *Arabidopsis*, switchgrass, and poplar biomass, supporting a role for pectin in biomass recalcitrance [64]. The present study shows that *C. thermocellum* is unable to effectively degrade the RG-I component of pectin, making RG-I a potential target for improving the ability of *C. thermocellum* to effectively solubilize woody biomass.

## Materials and methods

### Populus biomass preparation

*Populus trichocarpa* genotype GW-9947 was selected as the woody reference biomass for CBI (The Center for Bioenergy Innovation). GW-9947 is an elite natural variant from the BESC (BioEnergy Science Center) top line candidates with high ethanol yields, low lignin, and high S/G lignin [18]. Trees were harvested, debarked, chipped, air dried and milled to a particle size of 20 mesh (0.85 mm). The ground samples were used for the described analyses.

### Consolidated bioprocessing (CBP)

#### *C. thermocelluminoculum culture medium, growth medium and growth conditions*

Fermentations were carried out using *C. thermocellum* strain DSM1313. Cultures were grown in 1 × MTC (Medium for Thermophilic Clostridia) solution. The MTC stock solutions were diluted with H_2_O and the desired carbon source to reach 1 × concentration. The medium was prepared as described in Holwerda and coworkers [65] with the only exception being that CBI reference poplar was used as the carbon source (5 g/L glucan loading) to replace Avicel™ or cellobiose. For seed train cultures, the same medium was used except that 5 g/L cellobiose was the primary carbon source for initial serum bottle seeds and 5 g/L Avicel™ was the primary carbon source for the seed bioreactors. All growth medium was prepared at pH 7.0 and maintained at this level for all bioreactor experiments.

For bioreactor preparation, a 2 × MTC solution was prepared and filter sterilized without the carbon source. This solution was placed in an anaerobic chamber under 85% N_2_, 10% CO_2_, and 5% H_2_ until the bioreactors for carrying out the fermentations were sterilized. This 2 × stock was then aseptically added to the autoclaved reactors already containing water and CBI reference poplar under a sterilized biological safety cabinet to bring the total volume to a 1 × MTC concentration.

Serum bottles were prepared by making a 1 × MTC stock that included cellobiose and allowing the solution to reach anaerobic conditions in the anaerobic chamber described above. The medium was then transferred to the desired serum bottle size inside the chamber, capped, and stored at 4 °C until ready for use. Serum bottle seeds were allowed to proliferate to an OD_600_ of approximately 0.6 before transferring at 10% v/v to the 500-mL Avicel™ seed reactors. Avicel™ seed reactors were grown for approximately 24 h before transferring at 10% v/v to the CBI poplar containing reactors. Avicel™ seed cultures were monitored for ideal transferring phase by following total base addition (2 M KOH) and visual confirmation of a healthy culture under a microscope.

### Fermentations

Six Sartorius vessels of 0.5 L total working volume were set up identically for fermentation. A total of 5.68 g of CBI poplar (80/20 mesh sieved) was loaded into each reactor to reach a glucan loading of 5 g/L (0.5% w/v). The reactor, biomass, and water were autoclaved for 30 min at 121 °C. MTC media components were then added post-autoclaving as described above. Cultures were controlled at pH 7.0 with 2 N HCl and 2 N KOH as the acid and base, respectively. Temperature was maintained at 60 °C, agitation at 150 rpm minimum to keep biomass from accumulating on the bottom of the reactor, and 50 ccm of N_2_ was sparged through the reactor headspace to maintain anaerobic conditions. All reactors were inoculated with a 10% (v/v) inoculum of DSM1313 *C. thermocellum* from a common seed culture containing 5 g/L Avicel™. At 24, 48, and 120 h, two reactors each were shut down and harvested (Additional file 1: Fig. S1). Similar reactors were set up to act as controls that were not inoculated with *C. thermocellum* and instead brought up to the final volume with medium.

#### *Separations and quantification of total biomass solubilization*

To separate the undigested solids from the liquor, the reactor contents were split into 250-mL bottles and centrifuged at 10,000 RPM for 15 min in a Sorvall RC 6 Plus with FiberliteTM F14-6 × 250y fixed angle rotor. The supernatant was removed by pipetting, frozen for storage, and this fraction was referred to as ‘liquor’. The solids residue was washed with distilled water at a volume consistent with the liquor removed and centrifuged again under the same conditions. The wash fraction was removed by pipetting and the residual solids were dried for 5 days at 60 °C. The dried biomass was weighed, and the total solids solubilization was calculated by dividing the total recovered biomass after fermentation by the initial biomass loaded into the reactors at the start of fermentation.

Specifically, the following calculations were used:

Mass of polymer solubilized during control fermentation (without microbe) = *A*;

*A* = mass (mg) of sugar polymer in starting biomass before CBP minus the mass of solids in 24 h, 48 h, and 120 h fermentation control;

Mass of polymer solubilized during the CBP process (i.e., solubilized by the process conditions plus solubilization by *C. thermocellum*) = *B*;

*B* = mass (mg) of polymer in starting biomass before CBP minus the mass of solids in 24 h, 48 h, and 120 h CBP fermentations);

Mass of polymer solubilized by *C. thermocellum* = *B* − *A*;

$$\text{Percentage}\;({\%})\;\text{of}\;\text{polymer}\;\text{solubilization}\;\text{by}\;C.\; thermocellum =\frac{B-A}{\text{Starting}\;\text{biomass}} \times 100.$$

### Cell wall analysis

Alcohol insoluble residue (AIR) from solid residues post-fermentation was prepared as previously described [4, 17]. Prior to the analysis, approximately 40 mg of AIR samples were treated with alpha-amylase (0.47 U per mg biomass, Sigma Cat # A6255) in 4 mL of 100 mM ammonium formate (pH 6.0) at 25 °C for 48 h to remove starch, followed by three ddH_2_O and two acetone washes. The AIR samples were then kept in fume hood for 72 h to dry. Glycosyl residue composition analysis of the AIR (~ 2 mg) and liquor (100–300 µg) was by GC–MS of trimethylsilyl (TMS) derivatization of the monosaccharide methyl glycosides produced from the sample by acidic methanolysis as previously described [17, 62, 66]. Briefly, the samples were aliquoted into individual tubes and inositol (20 μg) was added as an internal standard. The samples were hydrolyzed for 18 h at 80 °C in 400 μL 1 M methanolic–HCl and the walls were then derivatized with 200 μL of TriSil and heated to 80 °C for 20 min. After filtering through packed glass wool, the dried samples were resuspended in 150 μL hexane and 1 μL of sample was injected into the GC–MS as described earlier [17].

### Saeman hydrolysis method of cellulose quantification

One method to determine cellulose content was to first quantify the total glucose content of the sample by Saeman hydrolysis of the cellulose in the sample [21, 22] followed by glycosyl residue composition analysis to give the total glucose (Glc) content and then subtracting from that the non-cellulosic glucose content as determined by TMS GC–MS (described above). The Saeman hydrolysis method hydrolyzes crystalline cellulose. Specifically, around 2 mg of AIR solid residue with 20 μg of inositol internal standard was hydrolyzed in 500 μL of 72% H_2_SO_4_ a borosilicate glass screw-top test tube for 1 h with vortexing every 15 min at room temperature. Samples were diluted to 1 M H_2_SO_4_ and heated to 121 °C for 1 h. Cooled samples were neutralized with 0.25 M Ba(OH)_2_. Supernatants were collected after centrifugation at 3600×*g* for 20 min and dried down by lyophilization. The quantification of glucose in the Saeman hydrolysis sample was by the GC–MS of TMS-derivatized methyl glycosides as described above [17]. This value represented the total cellulosic plus non-cellulosic Glc in the sample. The amount of fibrillary cellulose was obtained by subtracting the amount of non-cellulosic Glc obtained from a separate TMS GC–MS composition analysis from the total Glc value to give the total cellulose content of the sample.

### Methyl alditol (MA) glycosyl residue composition method for cellulose quantification

Glycosyl residue composition analysis was performed by GC–MS of the per-*O*-methyl alditol (MA) derivatives of the monosaccharides produced from the sample as described previously by Black and coworkers [25]. Permethylation of the AIR samples (~ 100 μg) was by two rounds of treatment with 400 μL sodium hydroxide (15 min) and 100 μL methyl iodide (45 min). After extraction in DCM (dichloromethane), the samples were hydrolyzed using 2 M TFA (2 h in sealed tube at 121 °C), reduced with NaBD_4_, and re-permethylated as before. The resulting MAs were analyzed on an Agilent 7890A GC interfaced to a 5975C MSD (mass selective detector, electron impact ionization mode); separation was performed on a 30 m Supelco Equity-1 bonded phase fused silica capillary column. The value of Glc obtained from the MA method represented the total Glc content of the sample. The Glc attributed to cellulose was calculated by subtracting the non-cellulosic Glc content as determined by TMS GC–MS (described above) from the total Glc content to give the cellulose content.

### Lignin

The Complex Carbohydrate Research Center (CCRC) high-throughput pyrolysis molecular beam mass spectrometry (MBMS) method [67] was used to quantify the lignin content and S/G lignin monomer ratio of the solid residues post-fermentation that remained after CBP including the fermentation control. The samples were prepared in duplicate by weighing 1.5–3.0 mg into a stainless metal cup, which was single-shot pyrolyzed (Frontier Lab) at 500 °C to produce volatile compounds. The volatile compounds were analyzed for lignin using a molecular beam mass spectrometer (Extrel Core Mass Spectrometers). The raw data were processed through UnscramblerX 10.1 software to obtain the principal components and raw lignin data. NIST 8492 (26.2% lignin content) and Aspen standards were also pyrolyzed and analyzed in the same manner and in the same batch as the unknown samples. Both standards were used for data quality control. Additionally, NIST 8492 was used to correct the raw lignin data.

### Preparation of cell wall fractions and total sugar estimation

Sequential fractionations of AIR from post-fermentation solid residues were carried out to isolate fractions enriched for different types of cell wall components as previously described [4, 62]. For the enzyme-linked immunosorbent assays,  AIR (200 mg) wall was suspended in 20 mL of 50 mM ammonium oxalate, pH 5.0 at 25 °C for 24 h with constant shaking at 100 rpm. After the incubation, the mixture was centrifuged at 4000*g* for 15 min at room temperature and the supernatant removed and named as the ammonium oxalate (AO) extract. The residual pellet was subsequently washed three times with the same volume of deionized water, centrifuged, and the supernatant discarded. The pellet was suspended in 20 mL of 4 M KOH with 1% (w/v) sodium borohydride and incubated at 25 °C for 24 h with constant shaking at 100 rpm. After incubation, the suspension was centrifuged at 4000*g* for 15 min and the collected supernatant labeled as 4 M KOH extract and stored at 4 °C. The 4 M KOH fraction was neutralized using glacial acetic acid. Both the AO and 4 M KOH fractions were dialyzed against six changes of deionized water at room temperature for 72 h and lyophilized.

Both the AO and 4 M KOH cell wall extracts were dissolved in deionized water at a concentration of 200 µg/mL. The amount of total sugar in both extracts was determined by the phenol–sulfuric acid assay as previously described [68, 69]. In brief, 100 μL of each cell wall extract was distributed into respective triplicate tubes and 100 μL of 5.0% (v/v) phenol was added to each tube. Subsequently, 500 μL of H_2_SO_4_ was added to each tube, followed by rapid vortexing to provide uniform mixing. The solutions were incubated for 20 min at room temperature in a fume hood. After incubation, 250 μL of each reaction mixture was added into the wells of a Costar 3598 ELISA plate and the absorbance was measured at 490 nm in a microplate reader (Bio-Rad). Standard curves were measured in triplicate with d-glucose as a standard to calculate the total sugar in the AO and 4 M KOH wall extracts.

### Enzyme-linked immunosorbent assay (ELISA) assay

The ammonium oxalate and 4 M KOH extract samples were applied (50 µL of 20 µg/mL) to 96-well plates and dried overnight at 37* °C* as described previously [27]*.* The plates were blocked with 200 µL of 1% (w/v) instant nonfat dry milk in 0.1 M Tris-buffered saline (TBS, pH 7.6) for 1 h at room temperature. The aspiration and wash steps were performed using an ELx405 microplate washer (Bio-Tek Instruments). Blocking agent was removed by aspiration, and 50 µL of primary monoclonal antibodies (mAbs) were added to each well and incubated for 1 h at room temperature. The aspiration of the primary antibodies was completed, and each well was washed three times with 300 µL of 0.1% (w/v) instant nonfat dry milk in Tris-buffered saline (wash buffer). After washing, 50 µL of secondary antibody, peroxidase-conjugated anti-mouse IgG whole molecule goat antibodies (diluted 1:5000 in wash buffer, Sigma-Aldrich Catalogue A4416), was added to each well and incubated at room temperature for 1 h. Wells were washed five times with 300 µL of wash buffer and 50 µL of TMB (3,3′,5,5′-tetramethylbenzidine) substrate solution was added to each well. After 20 min, the reaction was stopped by adding 50 µL of 0.5 N sulfuric acid. The net OD values of the color formation in the each well of the ELISA plates were measured at 450 nm and a background reading at 655 nm was measured and subtracted using a model 680 microplate reader (Bio-Rad). The primary antibodies used were the anti-RG-I CCRC-M14, CCRC-M35 and CCRC-M72 [26, 27] and the anti-xylan CCRC-M137, CCRC-M149, and CCRC-M152 [26–28].

## Supplementary Information

**Additional file 1**: **Fig. S1.** Schematic workflow of *C. thermocellum* consolidated bioprocessing (CBP) of milled un-pretreated poplar biomass. The specific amounts of biomass and water (200 mL) in fermentation vessels were autoclaved, after which 250 mL of filter sterilized 2 × MTC medium stock was added. CBP reactors were inoculated with 50 mL *C. thermocellum* culture from a common Avicel™-grown seed culture. Uninoculated controls (50 mL medium added instead) were included to account for non-biological solids solubilization. All fermentations were carried out at 60ºC and pH 7.0 with titration using 2 M KOH and with agitation at a minimum of 150 RPM to maintain solids suspended within the reactor. Sacrificial reactors were harvested in duplicate after 24, 48, and 120 h of fermentation, and the post-fermentation residual solids were separated from the fermentation liquor via centrifugation followed by drying of the solids at 60℃ for five days.

**Additional file 2**: **Fig. S2.** Total solids solubilization of CBI reference poplar biomass by *C. thermocellum* over 5 days at 60 °C. Solubilization profiles were generated by comparing post-fermentation dry weights to initial biomass loading (5.68 g). Mean ± standard deviation, n = 4. Statistical analysis was with one-way ANOVA followed by Fisher’s least significant difference method *P < 0.05, **P < 0.01

**Additional file 3: Fig. **S3. Glycosyl residue composition by trimethylsilyl (TMS) derivatization and GC–MS of sugars recovered in the liquor from the fermentations at the indicated times, as described in Fig. 3. The amounts of sugar are represented as average mol% (A), and mass of total monosaccharide quantified from total soluble liquor (B). Mean ± SD of two biological and two technical replicates (n = 4). Significant P values are expressed as *P < 0.05, **P < 0.001 at significant level (one-way ANOVA followed by Fisher’s least significant difference method).

**Additional file 4**: **Fig. S4.** Mass of AIR extracted per gram solid residue recovered after CBP of poplar biomass. n = 4, Statistical analysis was by one-way ANOVA followed by Fisher’s least significant difference method; *P < 0.05.

**Additional file 5**:** Fig. S5.** Total lignin, S, G, and H lignin of poplar biomass after CBP bioconversion with *C. thermocellum*. (**A**) Total lignin, (**B**) S lignin, (**C**) G lignin, and (**D**) H lignin content. Data are presented as % AIR prepared from solid residue after CBP. *P < 0.05, **P ≤ 0.01 significant level (one-way ANOVA followed by Fisher’s least significant difference method).

**Additional file 6**: **Fig. S6.** Data used to determine cellulose content of AIR from CBI reference poplar solid residues recovered over 120 h fermentation by *C. thermocellum* and from fermentation controls. (A-C) Cellulose content was estimated as the difference of (**A**) total cellulosic and non-cellulosic glucose of AIR pretreated by Saeman hydrolysis minus (**B**) the content of non-cellulosic glucose of AIR not treated by Saeman hydrolysis as detected by TMS derivatization and GC–MS. (**C**) The difference of glucose content between A and B is recognized as cellulose content and also presented in Fig. 5A. (D-F). Cellulose content was estimated as the difference of (**D**) total cellulosic and non-cellulosic glucose of AIR pretreated by methylated alditol method minus (**E**) the content of non-cellulosic glucose of AIR not treated by Methylated Alditol method as detected by TMS derivatization and GC–MS. (**F**) Cellulose content, the difference in glucose content between D and E, measured by Methylated Alditol method and also presented in Fig. 5B. The amounts of sugar are represented as average µg of glucose per mg of AIR. Mean ± SD, n = 6. P value is ** P ≤ 0.01 at significant level.

**Additional file 7**: **Fig. S7.** Glycosyl residue composition of AIR (e.g., cell walls) from solid residue recovered after CBP fermentation by trimethylsilyl (TMS) derivatization and GC–MS analysis. The amounts of sugar are represented as average of mol% (**A**), and mass (µg/mg AIR) of total monosaccharides in the solid residue (**B**). Mean ± SD of two biological and two technical replicates (n = 4). Stars indicate values that are different at * P ≤ 0.05, ** P ≤ 0.001 significant level.

## Data Availability

All data generated or analyzed during this study are included in the article/supplementary materials.

## Abbreviations

AIR: Alcohol-insoluble residue
Ara: Arabinose
CBP: Consolidated bioprocessing
Gal: Galactose
GalA: Galacturonic acid
GC–MS: Gas chromatography–mass spectrometry
Glc: Glucose
Man: Mannose
MA: Methylated alditol
MBMS: Molecular beam mass spectrometer
Rha: Rhamnose
RG-I: Rhamnogalacturonan I
TMS: Trimethylsilyl
Xyl: Xylose
