# Supplementary figures and images for: Composition and yield of non-cellulosic and cellulosic sugars in soluble and particulate fractions during consolidated bioprocessing of poplar biomass by Clostridium thermocellum
Source: Biotechnol Biofuels Bioprod. 2022 Feb 28;15:23. doi: 10.1186/s13068-022-02119-9 (PMC8887089; doi:10.1186/s13068-022-02119-9)

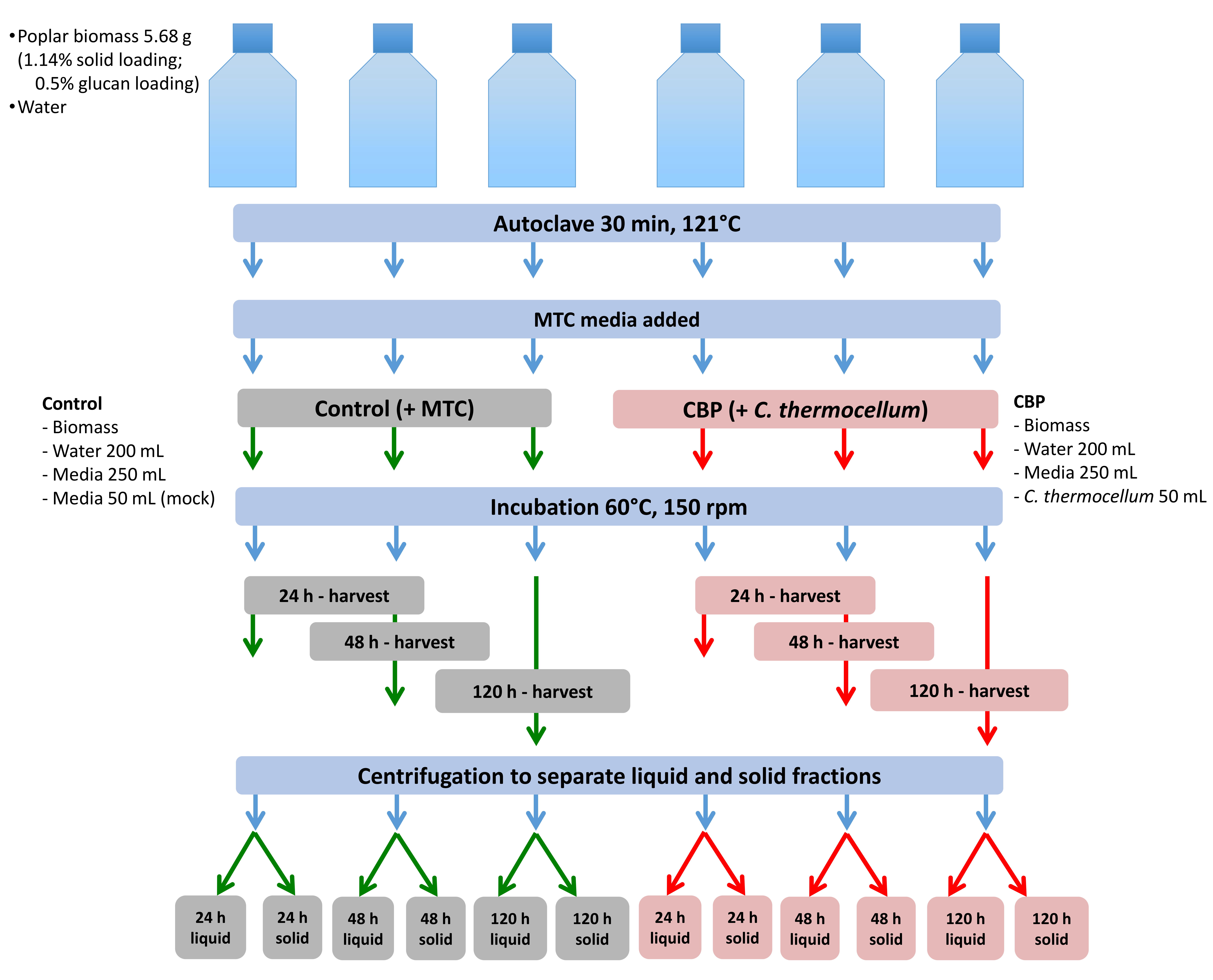

Supplement: Supplementary file 1 — Additional file 1: Fig. S1. Schematic workflow of C. thermocellum consolidated bioprocessing (CBP) of milled un-pretreated poplar biomass. The specific amounts of biomass and water (200 mL) in fermentation vessels were autoclaved, after which 250 mL of filter sterilized 2 × MTC medium stock was added. CBP reactors were inoculated with 50 mL C. thermocellum culture from a common Avicel™-grown seed culture. Uninoculated controls (50 mL medium added instead) were included to account for non-biological solids solubilization. All fermentations were carried out at 60ºC and pH 7.0 with titration using 2 M KOH and with agitation at a minimum of 150 RPM to maintain solids suspended within the reactor. Sacrificial reactors were harvested in duplicate after 24, 48, and 120 h of fermentation, and the post-fermentation residual solids were separated from the fermentation liquor via centrifugation followed by drying of the solids at 60℃ for five days. [file 13068_2022_2119_MOESM1_ESM.tif]

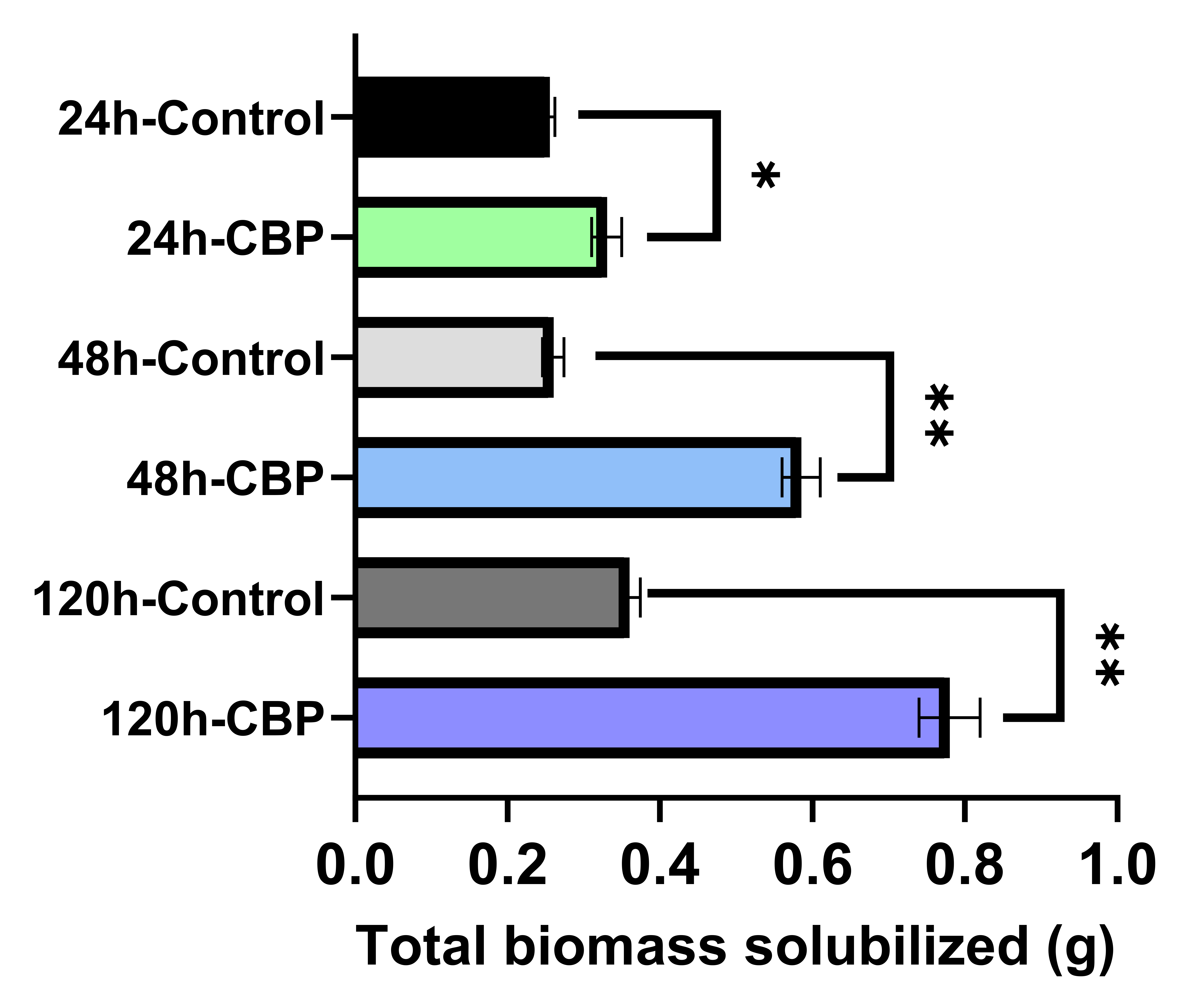

Supplement: Supplementary file 2 — Additional file 2: Fig. S2. Total solids solubilization of CBI reference poplar biomass by C. thermocellum over 5 days at 60 °C. Solubilization profiles were generated by comparing post-fermentation dry weights to initial biomass loading (5.68 g). Mean ± standard deviation, n = 4. Statistical analysis was with one-way ANOVA followed by Fisher’s least significant difference method *P < 0.05, **P < 0.01 [file 13068_2022_2119_MOESM2_ESM.tif]

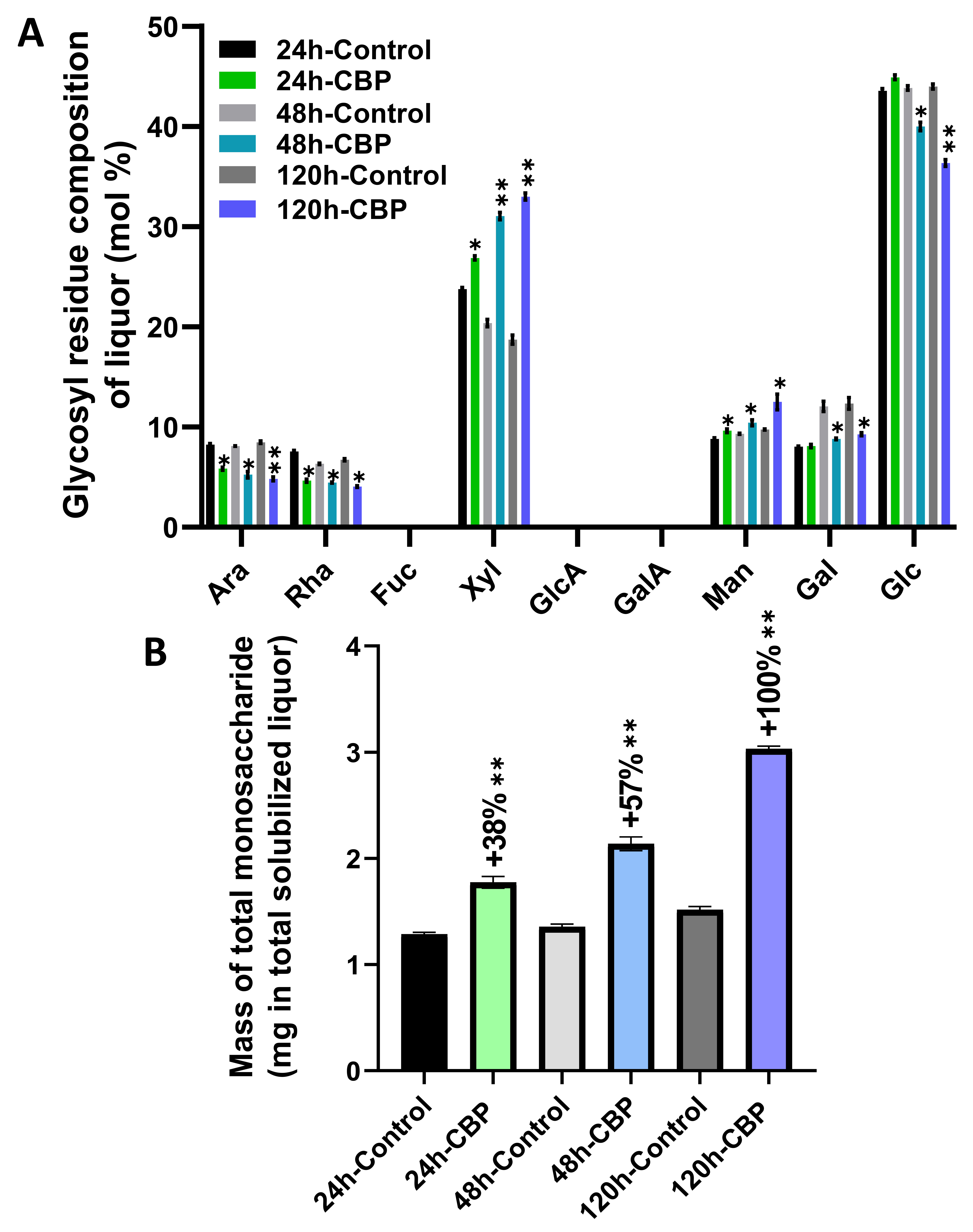

Supplement: Supplementary file 3 — Additional file 3: Fig. S3. Glycosyl residue composition by trimethylsilyl (TMS) derivatization and GC–MS of sugars recovered in the liquor from the fermentations at the indicated times, as described in Fig. 3. The amounts of sugar are represented as average mol% (A), and mass of total monosaccharide quantified from total soluble liquor (B). Mean ± SD of two biological and two technical replicates (n = 4). Significant P values are expressed as *P < 0.05, **P < 0.001 at significant level (one-way ANOVA followed by Fisher’s least significant difference method). [file 13068_2022_2119_MOESM3_ESM.tif]

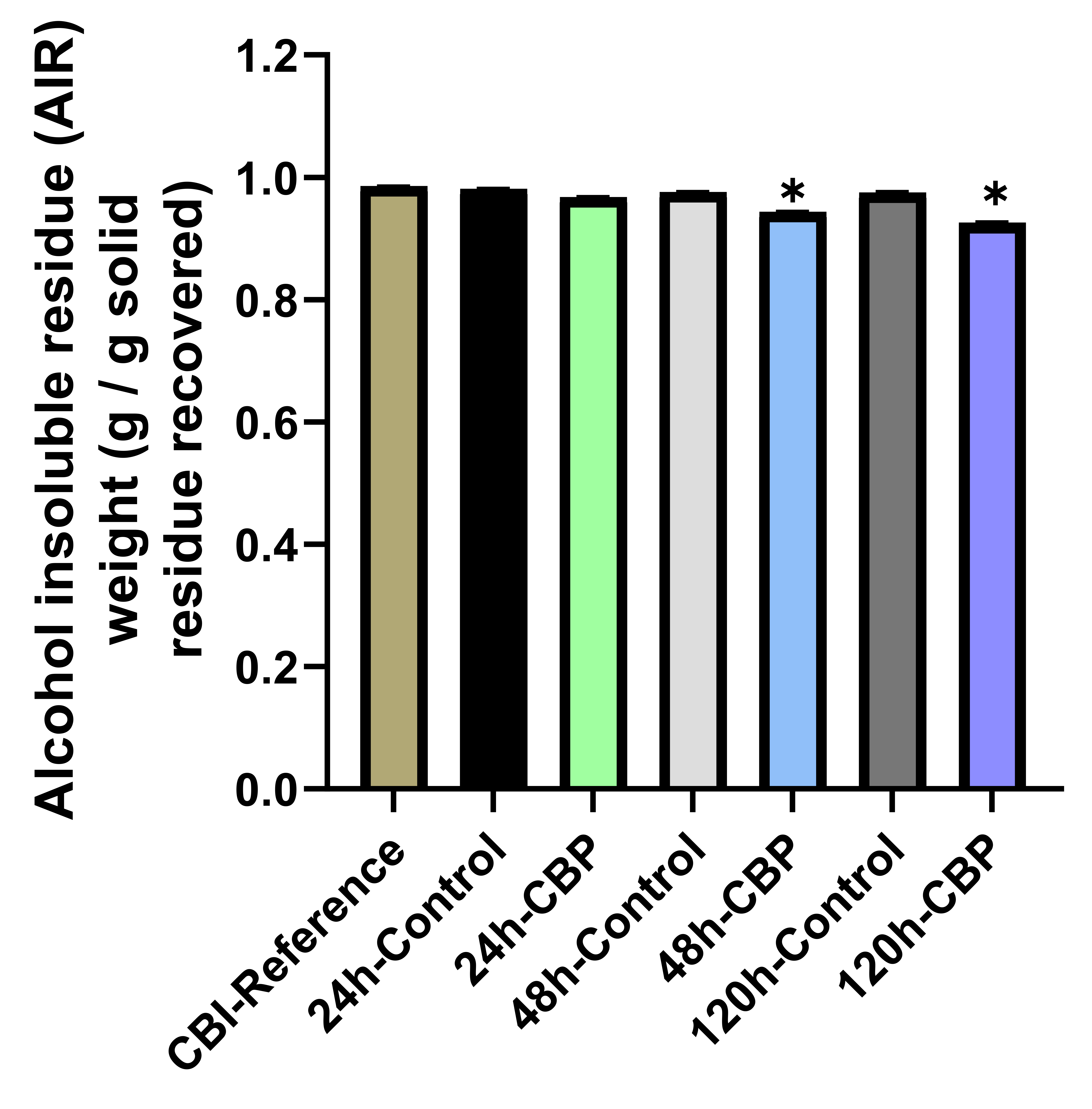

Supplement: Supplementary file 4 — Additional file 4: Fig. S4. Mass of AIR extracted per gram solid residue recovered after CBP of poplar biomass. n = 4, Statistical analysis was by one-way ANOVA followed by Fisher’s least significant difference method; *P < 0.05. [file 13068_2022_2119_MOESM4_ESM.tif]

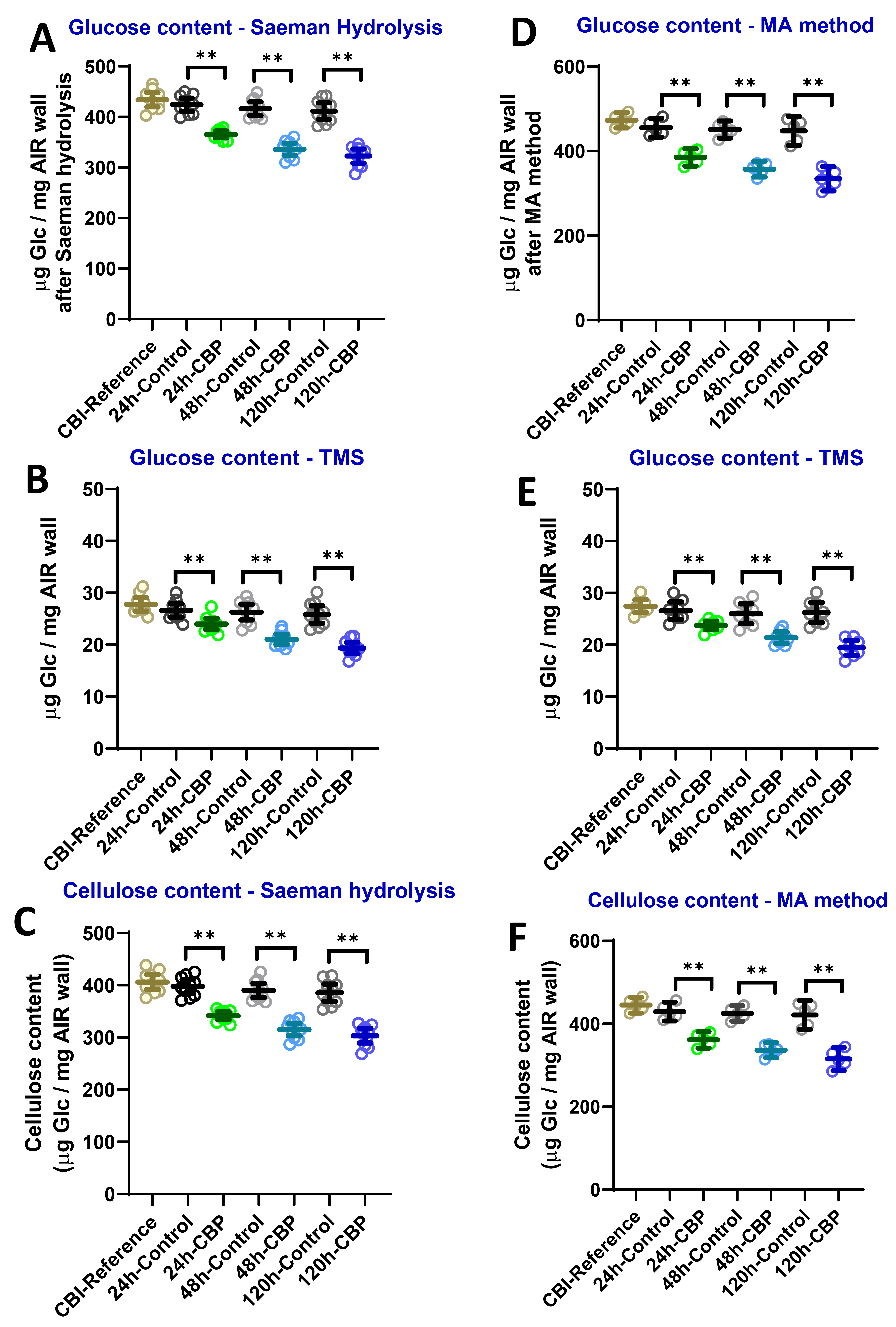

Supplement: Supplementary file 6 — Additional file 6: Fig. S6. Data used to determine cellulose content of AIR from CBI reference poplar solid residues recovered over 120 h fermentation by C. thermocellum and from fermentation controls. (A-C) Cellulose content was estimated as the difference of (A) total cellulosic and non-cellulosic glucose of AIR pretreated by Saeman hydrolysis minus (B) the content of non-cellulosic glucose of AIR not treated by Saeman hydrolysis as detected by TMS derivatization and GC–MS. (C) The difference of glucose content between A and B is recognized as cellulose content and also presented in Fig. 5A. (D-F). Cellulose content was estimated as the difference of (D) total cellulosic and non-cellulosic glucose of AIR pretreated by methylated alditol method minus (E) the content of non-cellulosic glucose of AIR not treated by Methylated Alditol method as detected by TMS derivatization and GC–MS. (F) Cellulose content, the difference in glucose content between D and E, measured by Methylated Alditol method and also presented in Fig. 5B. The amounts of sugar are represented as average µg of glucose per mg of AIR. Mean ± SD, n = 6. P value is ** P ≤ 0.01 at significant level. [file 13068_2022_2119_MOESM6_ESM.tif]

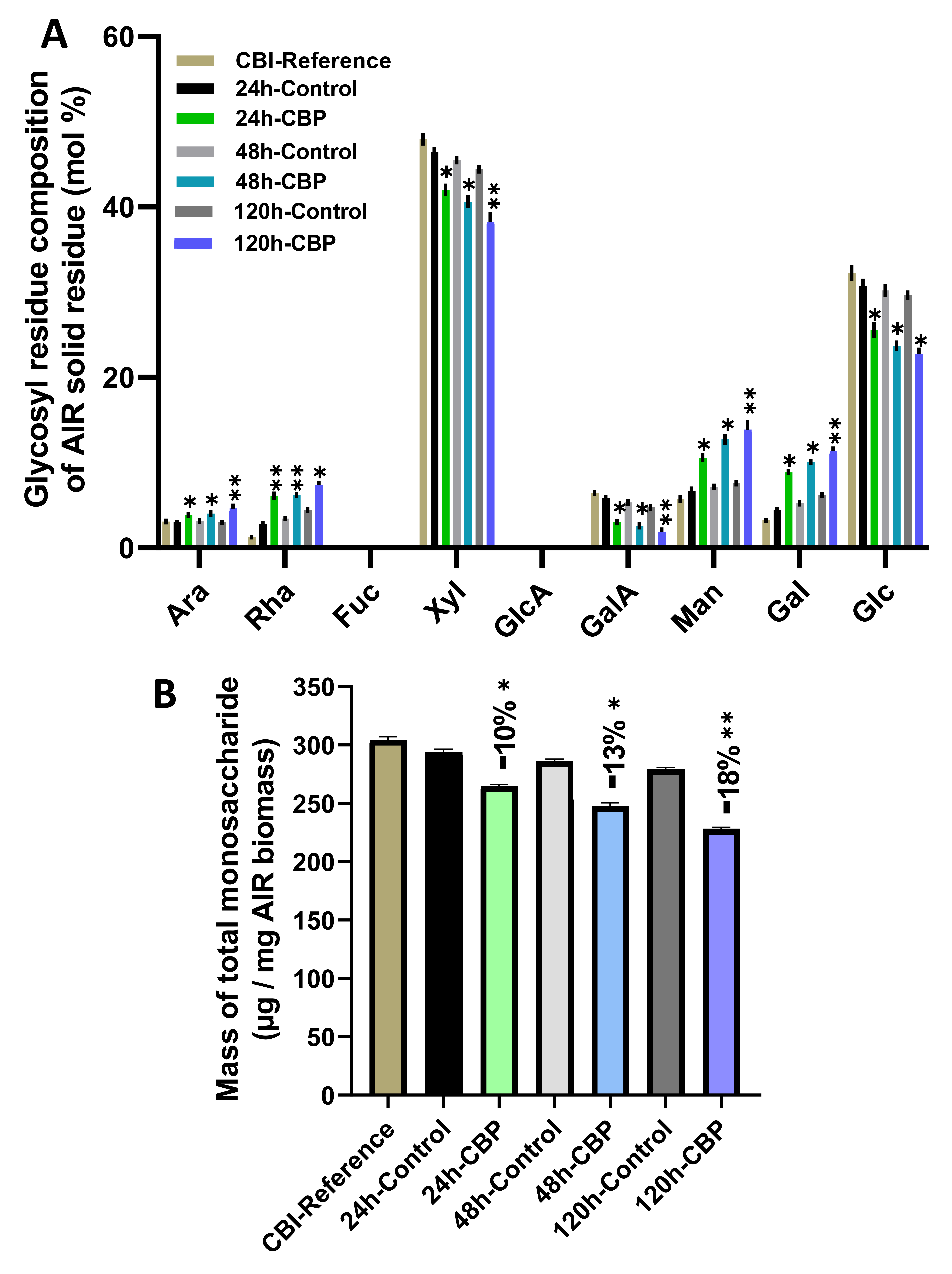

Supplement: Supplementary file 7 — Additional file 7: Fig. S7. Glycosyl residue composition of AIR (e.g., cell walls) from solid residue recovered after CBP fermentation by trimethylsilyl (TMS) derivatization and GC–MS analysis. The amounts of sugar are represented as average of mol% (A), and mass (µg/mg AIR) of total monosaccharides in the solid residue (B). Mean ± SD of two biological and two technical replicates (n = 4). Stars indicate values that are different at * P ≤ 0.05, ** P ≤ 0.001 significant level. [file 13068_2022_2119_MOESM7_ESM.tif]
